# The conserved helicase ZNFX-1 memorializes silenced RNAs in perinuclear condensates

**DOI:** 10.1038/s41556-022-00940-w

**Published:** 2022-06-23

**Authors:** John Paul Tsu Ouyang, Wenyan Lucy Zhang, Geraldine Seydoux

**Affiliations:** HHMI and Department of Molecular Biology and Genetics, Johns Hopkins University School of Medicine, Baltimore, MD USA

**Keywords:** RNAi, Caenorhabditis elegans, Epigenetics

## Abstract

RNA-mediated interference (RNAi) is a conserved mechanism that uses small RNAs (sRNAs) to silence gene expression. In the *Caenorhabditis elegans* germline, transcripts targeted by sRNAs are used as templates for sRNA amplification to propagate silencing into the next generation. Here we show that RNAi leads to heritable changes in the distribution of nascent and mature transcripts that correlate with two parallel sRNA amplification loops. The first loop, dependent on the nuclear Argonaute HRDE-1, targets nascent transcripts and reduces but does not eliminate productive transcription at the locus. The second loop, dependent on the conserved helicase ZNFX-1, targets mature transcripts and concentrates them in perinuclear condensates. ZNFX-1 interacts with sRNA-targeted transcripts that have acquired poly(UG) tails and is required to sustain pUGylation and robust sRNA amplification in the inheriting generation. By maintaining a pool of transcripts for amplification, ZNFX-1 prevents premature extinction of the RNAi response and extends silencing into the next generation.

## Abstract (web summary)

Ouyang et al. show that the RNA helicase ZNFX-1 preserves heritable RNAi by maintaining a pool of small RNA-targeted transcripts in perinuclear condensates of the *Caenorhabditis* *elegans* germline, which serve as templates for small-RNA amplification in the next generation.

## Main

RNA-mediated interference (RNAi) is a widespread mechanism that employs small RNAs (sRNAs) to modulate gene expression^1^. Core to the RNAi machinery are RNA-induced silencing complexes (RISC) consisting of single-stranded RNA, approximately 20 bases in length, complexed with Argonaute proteins. The RISC complexes recognize complementary RNAs and effect silencing by reducing RNA stability and/or translation efficiency^2–4^. Certain RISC complexes also recognize nascent transcripts and interfere with productive transcription by stalling RNA polymerase, RNA processing and/or recruiting chromatin modifiers to the locus^3,5,6^. In many organisms, sRNA pathways depend on cycles that amplify the production of sRNAs to achieve maximal silencing^3,7^. In *Drosophila*, a complex ‘ping-pong’ cycle in perinuclear condensates amplifies the processing of genomically encoded precursor transcripts containing sRNAs that target active transposable elements (PIWI-interacting RNA, piRNA)^7^. In *Schizosaccharomyces pombe*, the nuclear RISC-like complex (RITS) recruits an RNA-dependent RNA polymerase (RdRP) to the targeted locus^8^. The RdRP uses nascent transcripts as templates for continued synthesis of sRNAs that feed back into RITS^8^. In both cases, the sRNA amplification loops depend on transcription of the locus targeted for silencing to supply the template necessary to stimulate the processing (*Drosophila*) or de novo synthesis (*S. pombe*) of the relevant sRNAs.

As in *S. pombe*, sRNA amplification in *Caenorhabditis elegans* involves the activity of RdRPs that synthesize new sRNAs on transcripts recognized by RISC complexes. Two amplification mechanisms have been described. The first mechanism involves ‘primary’ sRNAs derived from genomically encoded loci (for example, piRNAs) or double-stranded RNA (dsRNA) processed by Dicer^3^. Recognition by primary sRNAs, complexed with primary Argonautes (for example, RDE-1), leads to RNA cleavage by the endonuclease RDE-8 and tailing of the 5′ fragment by the poly(UG) polymerase RDE-3 (also known as MUT-2)^9,10^. The ‘pUG’ tail recruits RdRPs that synthesize ‘secondary’ sRNAs near the cleavage site^9^. The secondary sRNAs in turn associate with secondary Argonautes (WAGO proteins) to trigger the degradation of complementary transcripts in the cytoplasm by an unknown mechanism^11^. A second cycle depends on the nuclear-enriched Argonaute HRDE-1 (refs. ^12–15^). Similar to RITS in *S. pombe*, the HRDE-1 cycle recognizes nascent transcripts and coordinates sRNA synthesis and heterochromatin deposition^3,6,8^.

In *C. elegans*, the silenced state can be passed on to progeny not exposed to the initial trigger^1,6,16–18^. Progeny of worms exposed to dsRNA produce pUGylated transcripts, suggesting that pUGylation-dependent sRNA amplification is heritable^9,19^. An early study examining RNAi in somatic tissues suggested, however, that only primary Argonautes initiate sRNA amplification^20^; in contrast, secondary Argonautes only target cognate messenger RNA for degradation^20^. Subsequent studies showed that production of ‘tertiary’ sRNAs is allowed in the germline and depends on HRDE-1 and other nuclear RNAi factors^15^. Unlike secondary sRNAs that map near the site of the primary sRNA trigger, tertiary sRNAs map throughout the transcript, possibly because they are templated off nascent transcripts^15^. Factors that accumulate in perinuclear condensates (nuage) outside nuclei have also been implicated in RNAi inheritance, including the Argonaute WAGO-4 and the helicase ZNFX-1 (refs. ^21–23^). Whether these factors function in the HRDE-1 cycle or a different sRNA amplification cycle has not yet been reported.

In this study, we examined the fate of germline mRNAs in animals exposed (by feeding) to a gene-specific dsRNA trigger. Our findings indicate that the HRDE-1 cycle of sRNA amplification, although sufficient to partially silence the locus, is insufficient for robust inheritance of the silenced state. A second cycle involving the Z granule-component ZNFX-1 is also required in parallel. We find that ZNFX-1 is responsible for localization of targeted mRNAs to perinuclear condensates, and to maintain pUGylation and the bulk of sRNA amplification in progeny.

## Results

### Targeted transcripts exhibit changes within 4 hours of RNAi

**Fig. 1:**
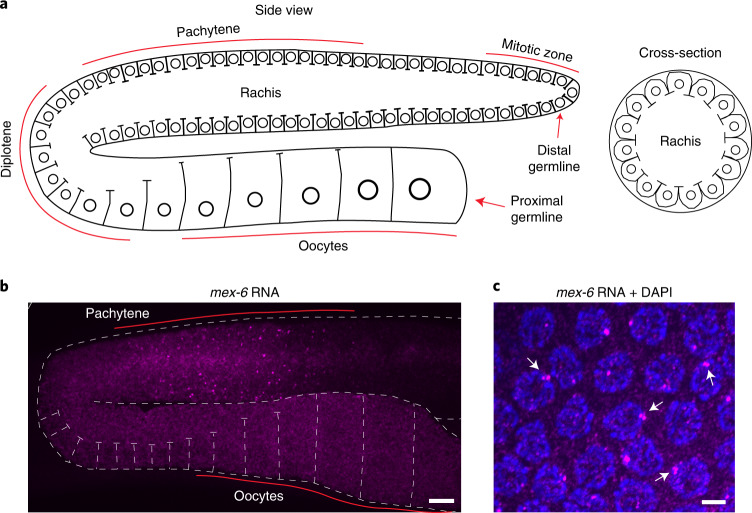
Expression of *mex-6* RNA in the *C. elegans* germline. **a**, Schematics (adapted from^62^) depicting the adult hermaphrodite germline. Circles indicate germline nuclei; lines indicate plasma membranes. Nuclei at the distal end are in mitosis and progress through stages of meiotic prophase (pachytene and diplotene) and oogenesis as they move towards the proximal end (left). The cross-sectional (right) view shows the pachytene region. A common cytoplasm (rachis) runs through the entire germline, except for the most proximal (mature) oocyte. Sperm are generated during larval development and stored in the spermatheca (not shown here). **b**, Maximum projection photomicrograph of an adult germline oriented as in **a** and hybridized to a *mex-6* antisense probe visualized using FISH hybridization (magenta). Scale bar, 10 µm. Image is representative of eight worms examined. Results were consistent across four independent FISH experiments. **c**, High-resolution photomicrograph showing pachytene nuclei (blue, stained with DAPI) and *mex-6* RNA (magenta). At this stage, homologous chromosomes are replicated and tightly synapsed at the nuclear periphery. Nascent transcripts at the *mex-6* locus are visualized as bright puncta that occasionally resolve into two or more closely apposed foci (arrows). Scale bar, 2.5 µm. Image is representative of three worms examined. Results were consistent across four independent FISH experiments.

**Extended Data Fig. 1:**
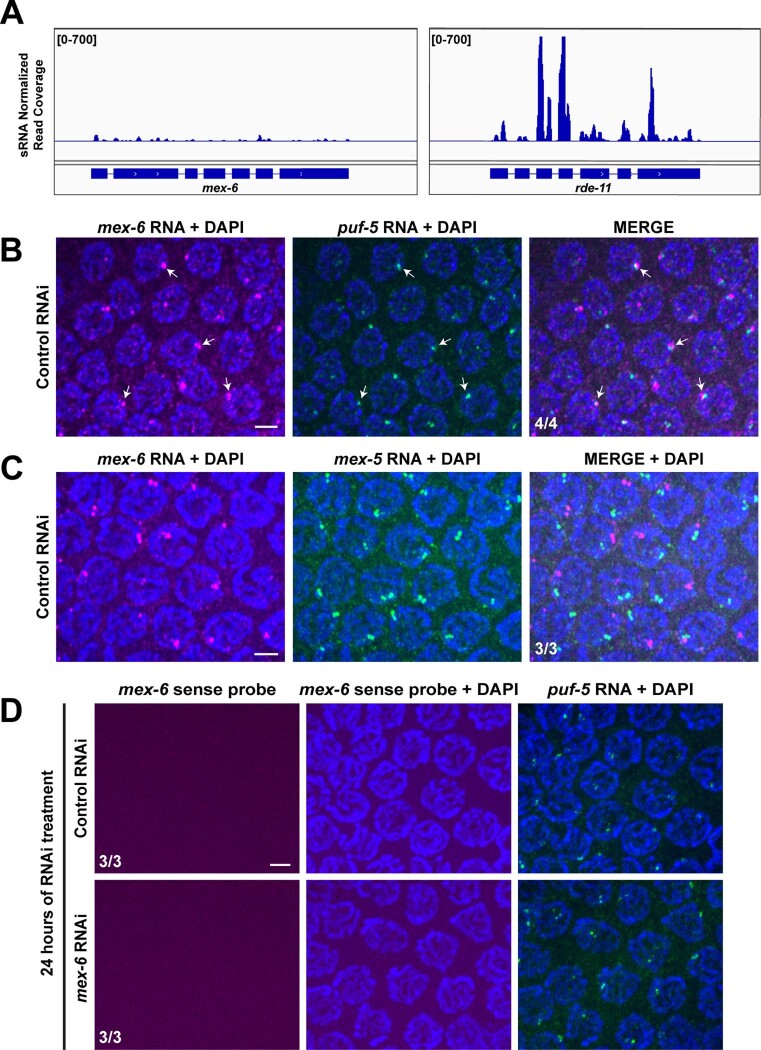
Characterization of the *mex-6* transcript. **a**) IGV genome browser views of sRNAseq reads (wild-type adult hermaphrodites) mapping to the *mex-6* locus. *rde-11* is a locus that is highly targeted by endogenous sRNAs^52^, shown here for comparison. Genome views are representative of two independent sequencing libraries. **b**) Maximum projection photomicrographs of pachytene nuclei showing DNA (blue, stained with DAPI), *mex-6* RNA (magenta), and *puf-5* RNA (green). The *mex-6* and *puf-5* loci are linked on Chromosome II and, as expected, exhibit closely linked nuclear puncta (arrows). Scale bar is 2.5 µm. Images are representative of 3 worms examined. Results were consistent across four independent FISH experiments. **c**) Same as in B but showing *mex-6* (magenta) and *mex-5* (green) RNAs. *mex-6* and *mex-5* are homologous loci on different chromosomes. Scale bar is 2.5 µm. Images are representative of 3 worms examined. Results were consistent across two independent FISH experiments. The *mex-6* and *mex-5* puncta do not co-localize, as expected, confirming the specificity of the FISH probes. **d**) Same as in B and C but using a *mex-6* sense probe (magenta) and a *puf-5* antisense probe (green). Scale bar is 2.5 µm. Images are representative of 3 worms examined. Results were consistent across two independent FISH experiments. As expected, the *mex-6* sense probe does not detect any signal, confirming that the signals detected by the *mex-6* antisense probe in panels B and C correspond to RNA and not DNA. Note also the lack of signal under RNAi conditions, suggesting that our FISH protocol does not detect sRNAs.

**Extended Data Fig. 2:**
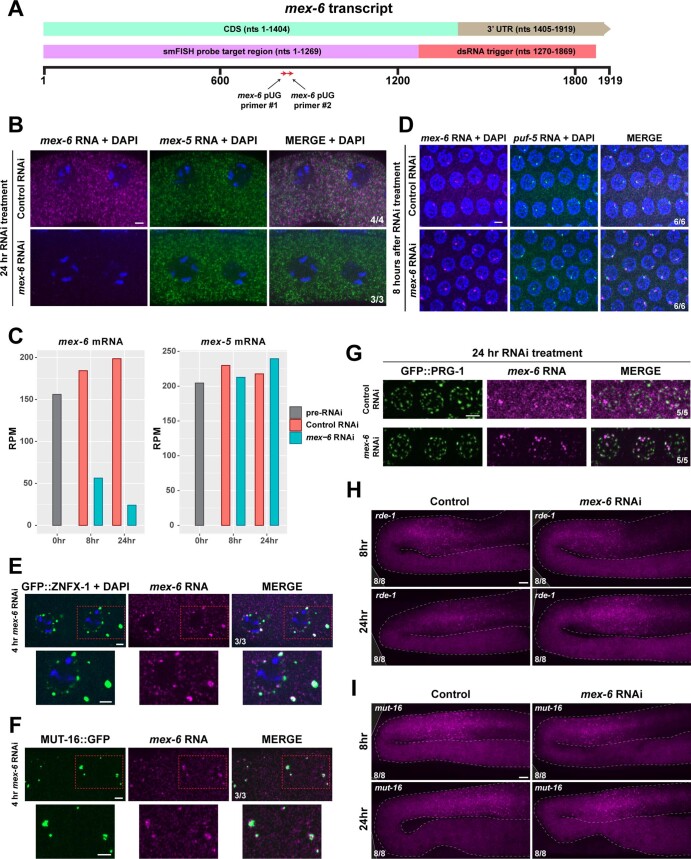
*mex-6* RNAi induces cytoplasmic and nuclear changes to the *mex-6* transcript, dependent upon the RNAi machinery. **a**) Schematic of the *mex-6* transcript. The regions targeted by smFISH probes and the dsRNA trigger are indicated. Red arrows denote primers used for amplification of pUGylated transcripts shown in Fig. 6. **b**) FISH experiment showing that *mex-6* RNAi conditions reduce *mex-6* but not *mex-5* levels in oocytes. Scale bar is 2.5 µm. Number of worms examined is indicated. Results were consistent across two independent FISH experiments. **c**) Control RNAseq experiment on adult hermaphrodites showing that *mex-6* RNAi conditions reduce *mex-6* but not *mex-5* transcripts. *mex-6* RPM counts exclude the trigger region. Each bar represents the *mex-6/mex-5* RPM of a single biological replicate. **d**) Control FISH experiment showing near co-localization of *mex-6* and *puf-5* signals under control and *mex-6* RNAi conditions. Scale bar is 2.5 µm. Images are representative of 6 worms examined. Results were consistent across four independent FISH experiments. **e**) Photomicrographs of oocytes showing the Z granule marker GFP::ZNFX-1, DNA, and *mex-6* RNA 4 h post *mex-6* RNAi treatment. Scale bar is 2.5 µm. Images are representative of 3 worms examined. Results were consistent across two independent FISH experiments. **f**) Photomicrographs of oocytes showing the Mutator foci marker MUT-16::GFP and *mex-6* RNA 4 h post *mex-6* RNAi treatment. Scale bar is 2.5 µm. Images are representative of 3 worms examined. Results were consistent across three independent FISH experiments. **g**) Photomicrographs of pachytene nuclei showing GFP::PRG-1 and *mex-6* RNA 24 h post either control or *mex-6* RNAi treatment. Scale bar is 2.5 µm. Images are representative of 5 worms examined. Results were consistent across four independent FISH experiments. **h**) Photomicrographs of *rde-1* mutant germlines showing *mex-6* RNA in either control or *mex-6* RNAi conditions at 8 and 24 h of RNAi treatment. Scale bar is 10 µm. Images are representative of 8 worms examined. Results were consistent across two independent FISH experiments. **i**) Photomicrographs of *mut-16* mutant germlines showing *mex-6* RNA in either control or *mex-6* RNAi conditions at 8 and 24 h of RNAi treatment. Scale bar is 10 µm. Images are representative of 8 worms examined. Results were consistent across two independent FISH experiments. Source data

**Extended Data Fig. 3:**
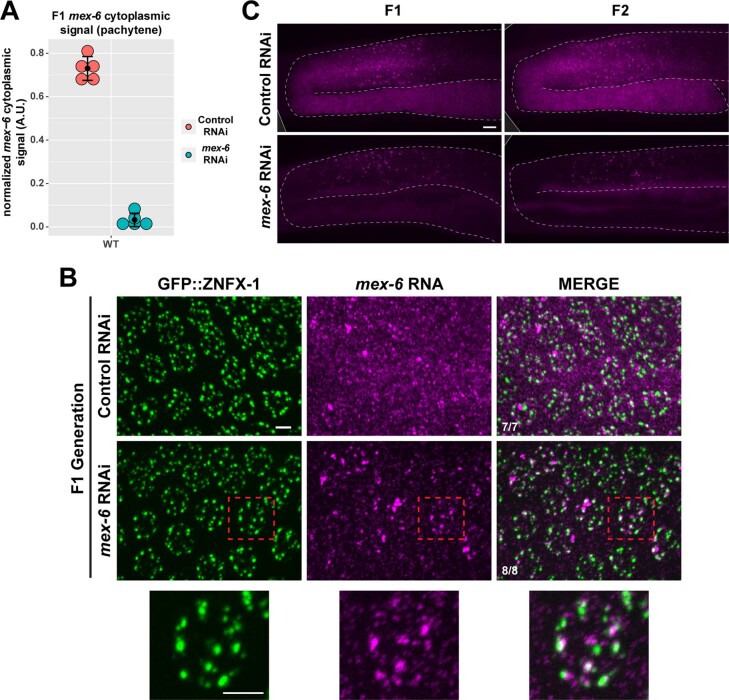
Transgenerational analysis of the *mex-6* transcript upon RNAi. **a**) Graph comparing the mean *mex-6* RNA FISH signal from the pachytene rachis in wild-type F1 progeny of P0s administered either control (red) or *mex-6* (blue) RNAi. Each dot represents a single worm (n = 5 worms). Central black dot and error bars represent the mean and standard deviation, respectively. Values (arbitrary units) were normalized to *puf-5* RNA FISH signals visualized in same region (Methods). **b**) Maximum projection photomicrographs of pachytene nuclei showing the nuage marker GFP::ZNFX-1 (green) and *mex-6* RNA (magenta) in F1 progeny of animals exposed to *mex-6* RNAi. Bottom rows shows high-resolution images of a single pachytene nucleus. Scale bar is 2.5 µm. Images are representative of 7 worms examined in the control condition and 8 worms examined in the *mex-6* RNAi condition. Results were consistent across two independent FISH experiments. **c**) Maximum projection photomicrographs showing *mex-6* RNA in germlines from F1 and F2 progeny derived from P0 animals exposed to *mex-6* or control RNAi conditions. Scale bar is 10 µm. Images are representative of 6 worms examined in each condition. This experiment was performed once. Source data

To examine the consequences of RNAi, we used fluorescent in situ hybridization (FISH) to visualize a model transcript expressed in the adult hermaphrodite germline (Fig. 1, Extended Data Fig. 1a and Methods). Similar to other maternal transcripts, *mex-6* RNA is transcribed in nuclei in the late pachytene region^24^ and accumulates in the shared cytoplasm (rachis) that supplies the growing oocytes^25^. As expected, we detected *mex-6* transcripts diffuse in the rachis and cytoplasm of growing oocytes and concentrated in bright nuclear puncta in pachytene nuclei (but not oocyte nuclei; Fig. 1b,c). At high magnification, the nuclear puncta overlapped with 4,6-diamidino-2-phenylindole (DAPI) staining and occasionally resolved into twin or triplet dots (Fig. 1c), consistent with tight pairing of replicated homologous chromosomes in pachytene nuclei^26^. The nuclear puncta represent nascent transcripts at the *mex-6* locus given that: (1) two-colour FISH targeting *mex-6* and a linked locus (*puf-5*, <1 Mb distance from *mex-6*) revealed closely linked puncta (Extended Data Fig. 1b), (2) two-colour FISH targeting *mex-6* and its non-linked homologue *mex-5* revealed well-separated puncta with no cross-hybridization (Extended Data Fig. 1c) and (3) *mex-6* nuclear puncta were only detected in the pachytene region and were not detected using a sense probe (Extended Data Fig. 1d).

**Fig. 2:**
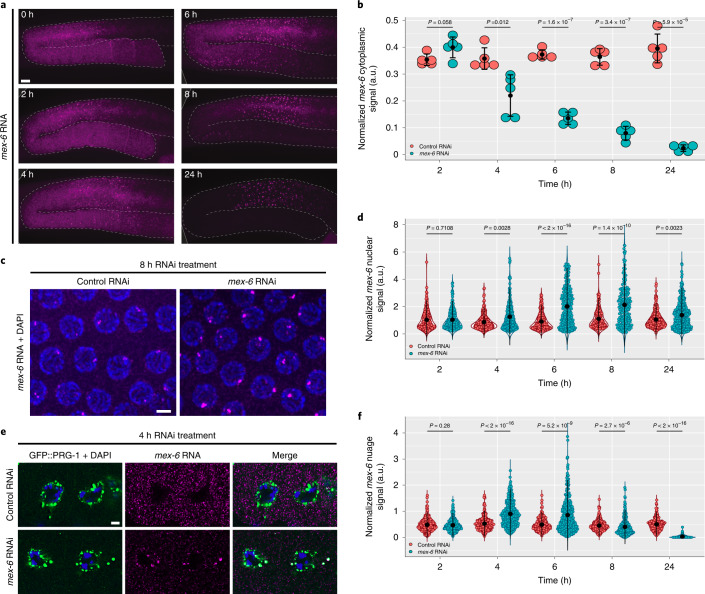
Evolution of *mex-6* RNA in P_0_ generation wild-type hermaphrodites over 24 h of RNAi treatment. **a**, Maximum projection photomicrographs of germlines oriented as in Fig. 1, with *mex-6* RNA (magenta) detected through FISH hybridization at the indicated times post the onset of RNAi feeding. Scale bar, 10 µm. Images are representative of eight worms examined for each condition. **b**, Comparison of the mean cytoplasmic the *mex-6* RNA FISH signals (diplotene region) in control and *mex-6* RNAi conditions at the indicated time points of RNAi treatment. Each dot represents a single germline (*n* = 5 germlines). **c**, Maximum projection photomicrographs of pachytene nuclei showing *mex-6* RNA (magenta) and DNA (blue, stained with DAPI) after 8 h of RNAi treatment. Scale bar, 2.5 µm. Images are representative of three worms examined for each condition. **d**, Comparison of maximum nuclear *mex-6* RNA FISH signals (pachytene region) in control and *mex-6* RNAi conditions at the indicated time points of RNAi treatment. Each dot represents one nucleus. Nuclei were quantified across three worms. **e**, Single *z*-plane photomicrographs of two oocytes stained for the nuage marker GFP::PRG-1 (green), DNA (DAPI; blue) and *mex-6* RNA (magenta) after 4 h of RNAi treatment. Scale bar, 2.5 µm. Images are representative of five worms examined for each condition. Results were consistent across three independent FISH experiments for **a**,**c**,**d**. **f**, Comparison of the average *mex-6* RNA FISH signal in nuage (diplotene) in control and *mex-6* RNAi conditions at the indicated time points of RNAi treatment. Each dot corresponds to a nuage granule. Nuage granules were quantified across five worms. **b**,**d**,**f**, Values were normalized to *puf-5* RNA FISH signals visualized in the same germline (**b**), nucleus (**d**) or nuage granule (**f**). The central black dot and error bars represent the mean and s.d., respectively. *P* values were calculated using an unpaired two-tailed Student’s *t*-test (**b**) or unpaired two-tailed Wilcoxon rank-sum test (**d**,**f**); a.u., arbitrary units. The exact number of nuclei (**d**) and nuage granules (**f**) quantified for each condition are provided in Source Data. Source data

To silence the *mex-6* gene, we exposed synchronized first-day adult hermaphrodites to a 600-base pair (bp) dsRNA trigger targeting the 3′ end of the *mex-6* transcript (Extended Data Fig. 2a and Methods). RNA sequencing (RNAseq) and two-colour FISH experiments confirmed that the RNAi treatment was specific for *mex-6* and did not affect *mex-5* levels (Extended Data Fig. 2b,c). We first detected a reduction in *mex-6* signal in the cytoplasm of diplotene oocytes after 4 h of RNAi treatment, culminating in >90% decrease by 24 h (Fig. 2a,b). We also detected a transient increase in the intensity distribution of *mex-6* pachytene nuclear puncta at 6 and 8 h (Fig. 2c,d). We still observed near co-localization of *mex-6* and *puf-5* nuclear puncta under *mex-6* RNAi conditions, confirming that the puncta identify nascent transcripts at the *mex-6* locus (Extended Data Fig. 2d). We conclude that in the first 24 h of exposure to the dsRNA trigger, RNAi induces a transient increase in the accumulation of nascent transcripts at the locus and a steady decrease in cytoplasmic transcripts.

### Targeted transcripts accumulate in nuage

From 4 h after the start of RNAi treatment, we also noticed accumulation of *mex-6* transcripts in micrometre-sized clusters in the cytoplasm of growing oocytes (Fig. 2a,e,f). The clusters overlapped with the P-granule marker PRG-1 and the Z granule-marker ZNFX-1, and overlapped partially with the Mutator foci-marker MUT-16 (Fig. 2e and Extended Data Fig. 2e,f). At 6 and 8 h, we also detected *mex-6* accumulation in perinuclear nuage in the diplotene and pachytene regions (Fig. 2a). At 24 h, *mex-6* accumulation in nuage in diplotene and growing oocytes was strongly diminished (Fig. 2f), mirroring the strong depletion of *mex-*6 transcripts in the cytoplasm (Fig. 2b). However, *mex-6* signal could still be detected in nuage in the pachytene region where the *mex-6* locus is transcribed (Extended Data Fig. 2g). We conclude that *mex-6* transcripts accumulate in nuage throughout the RNAi response. The resolution of the in situ experiments was not sufficient to determine whether *mex-6* RNA accumulates in a specific nuage subcompartment(s).

### RNAi-induced changes depend on the RNAi machinery and are heritable

**Fig. 3:**
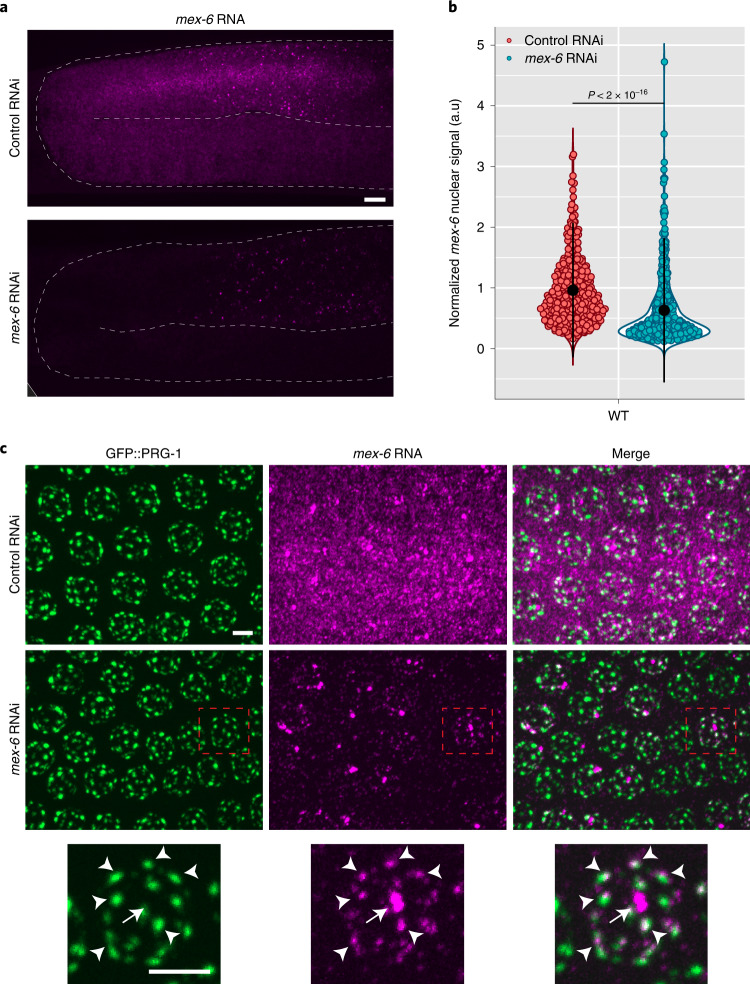
Levels of *mex-6* RNA in adult progeny (F_1_ generation) of animals exposed to *mex-6* dsRNA. **a**, Maximum projection photomicrographs of germlines showing *mex-6* RNA (magenta) in adult F_1_ Generation of animals exposed to control or *mex-6* RNAi (Methods). Scale bar, 10 µm. Images are representative of eight worms examined. **b**, Comparison of the maximum F_1_ *mex-6* nuclear signal (pachytene region) in the F_1_ progeny of animals exposed to control or *mex-6* RNAi. Each dot represents one nucleus. Nuclei were quantified across three worms (the exact number of nuclei quantified for each condition are provided in the Source Data). Values (arbitrary units, a.u.) were normalized to *puf-5* RNA FISH signals visualized in the same nuclei (Methods). The central black dot and error bars represent the mean and s.d., respectively. The *P* value was calculated using an unpaired two-tailed Wilcoxon rank-sum test. **c**, Maximum projection photomicrographs of pachytene nuclei showing the nuage marker GFP::PRG-1 (green) and *mex-6* RNA (magenta) in F_1_ Generation of animals exposed to control or *mex-6* RNAi. High-resolution images of a single pachytene nucleus (outlined by red boxes) are provided (bottom). The arrows point to *mex-6* RNA signal at the locus and the arrowheads point to *mex-6* RNA foci overlapping with perinuclear nuage. Scale bar, 2.5 µm. Images are representative of three worms examined. Note that the *mex-6* RNA signal overlaps but is not perfectly coincident with the P granule-marker PRG-1; *mex-6* RNA also partially overlaps with the Z granule-marker ZNFX-1, as shown in Extended Data Fig. 3b. Z granules are immediately adjacent to and/or overlap with P granules (within the diffraction limit) in pachytene and merge with P granules in embryos^22,63^. **a**,**c**, Results were consistent across three independent FISH experiments. Source data

RDE-1 is the Argonaute that recognizes primary sRNAs derived from exogenous triggers^11,27^ and MUT-16 is required for amplification of secondary sRNAs^28^. We found that *rde-1* and *mut-16* mutants were completely defective in the RNAi response (Extended Data Fig. 2h,i), confirming that the observed changes require synthesis of secondary sRNAs initiated by primary siRNAs. To examine inheritance of the response, we isolated embryos (F_1_ generation) from gravid hermaphrodites (P_0_ generation) fed the *mex-6* trigger and raised the F_1_ worms to the adult stage in the absence of the trigger. Cytoplasmic and nuclear *mex-6* RNA was strongly reduced in the F_1_ worms compared with the F_1_ controls (derived from P_0_ worms exposed to control RNAi; Fig. 3a,b and Extended Data Fig. 3a). Despite this strong reduction, we still detected transcripts in perinuclear dots overlapping with the nuage markers GFP::PRG-1 and GFP::ZNFX-1 in the pachytene region (Fig. 3c and Extended Data Fig. 3b). In contrast, little to no nuage accumulation was evident in oocytes (Fig. 3a). A similar pattern was detected in F_2_ animals (Extended Data Fig. 3c). These observations suggest that, despite a reduction in nascent transcripts, some *mex-6* transcripts are still exported from the nucleus and allowed to accumulate at least transiently in nuage in the pachytene region in F_1_ and F_2_ animals.

### *hrde-1* is required for nuclear RNAi response in P_0_ and F_1_ animals

**Fig. 4:**
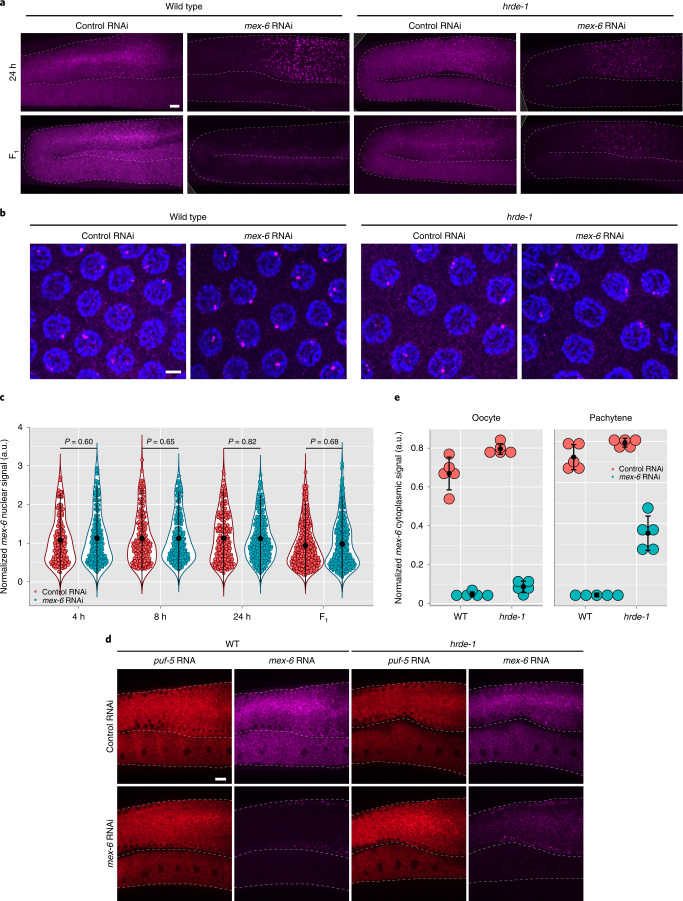
RNAi-induced changes in nascent transcripts require *hrde-1*. **a**, Maximum projection photomicrographs of germlines showing *mex-6* RNA (magenta) in P_0_ (24 h RNAi exposure; top) and F_1_ wild-type (bottom) and *hrde-1* mutants under control or *mex-6* RNAi conditions. Scale bar, 10 µm. Images are representative of eight worms examined for each condition. **b**, Maximum projection photomicrographs of pachytene nuclei in P_0_ wild-type and *hrde-1* mutant animals stained for *mex-6* RNA (magenta) and DNA (stained with DAPI; blue) following 8 h of either control or *mex-6* RNAi treatment. Scale bar, 2.5 µm. Images are representative of four worms examined for each condition. **c**, Comparison of the maximum nuclear *mex-6* RNA FISH signals (pachytene region) in P_0_ *hrde-1* mutants following either control or *mex-6* RNAi at the indicated time points. Each dot represents one nucleus. Nuclei were quantified across three worms (the exact number of nuclei quantified for each condition are provided in Source Data). *P* values were calculated using an unpaired two-tailed Wilcoxon rank-sum test. Refer to Fig. 2d for comparison to the wild type. **d**, Single *z*-plane photomicrographs showing *mex-6* RNA (magenta) and control *puf-5* RNA (red) in the cytoplasm in the pachytene and oocyte regions comparing *hrde-1* mutant and wild-type animals following 24 h of RNAi treatment. In wild-type worms, *mex-6* RNA is depleted in both regions but it is only partially depleted in the pachytene region of *hrde-1* mutants, consistent with a failure to silence the locus. Scale bar, 10 µm. Images are representative of eight worms examined for each condition. **a**,**b**,**d**, Results were consistent across three independent FISH experiments. **e**, Comparison of the mean *mex-6* RNA levels in the cytoplasm of oocytes and the pachytene region in *hrde-1* mutant and wild-type animals following 24 h of RNAi treatment. Each dot represents one animal (*n* = 5 worms). **c**,**e**, Values (arbitrary units, a.u.) were normalized to *puf-5* RNA FISH signals visualized in the same nuclei (**c**) or areas (**e**; Methods). The central black dot and error bars represent the mean and s.d., respectively. Source data

**Extended Data Fig. 4:**
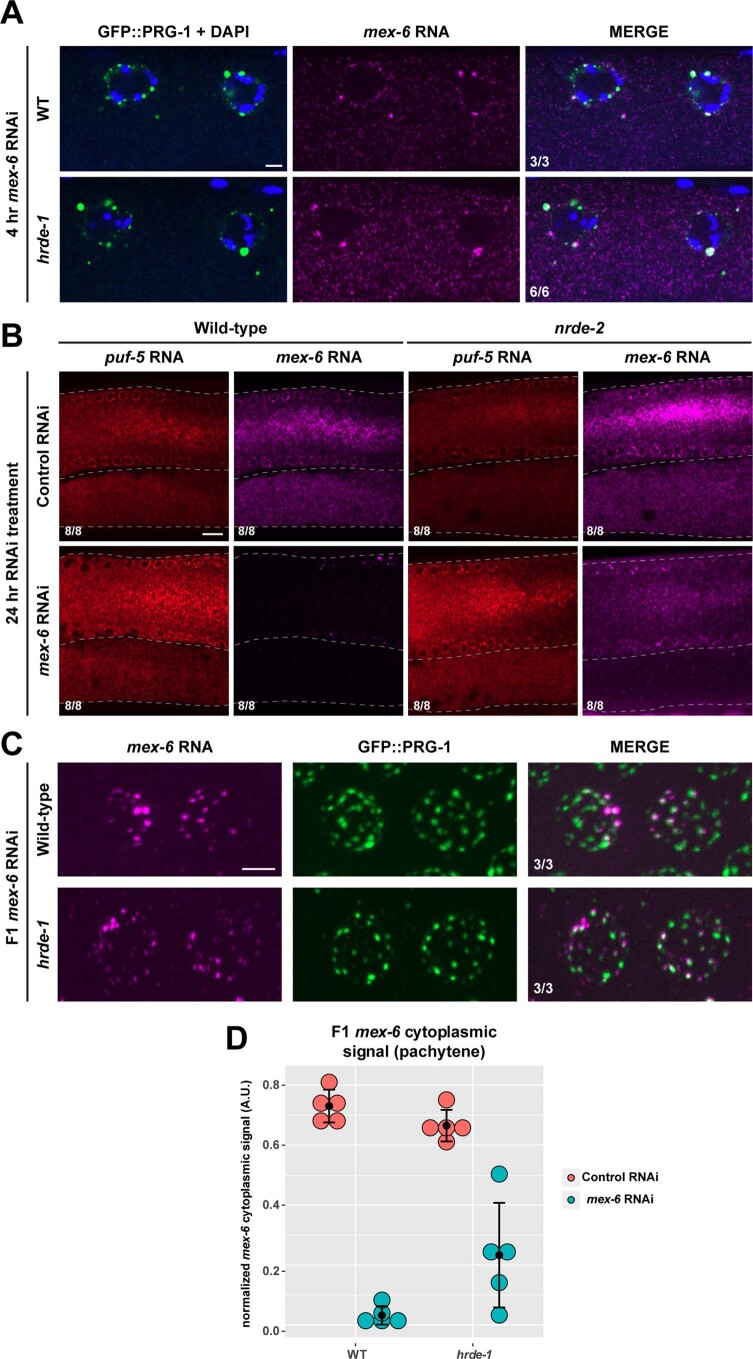
The nuclear RNAi pathway affects *mex-6* nascent transcripts upon RNAi. **a**) Photomicrographs of P0 wild-type and *hrde-1* mutant oocytes showing the nuage marker GFP::PRG-1 (green), DNA (blue, stained with DAPI), and *mex-6* RNA (magenta) following 4 h of *mex-6* RNAi treatment. Scale bar is 2.5 µm. Images are representative of 3 wild-type worms examined and 6 *hrde-1* worms examined. Results were consistent across three independent FISH experiments. **b**) Single z-plane photomicrographs of P0 wild-type and *nrde-2* mutant germlines (pachytene region and oocytes) at 24 h after control or *mex-6* RNAi co-stained for *mex-6* (magenta) and *puf-5* (red, negative control) RNAs. Scale bar is 10 µm. Images are representative of 8 worms examined in each condition. This experiment was performed once. **c**) Maximum projection photomicrographs of F1 wild-type or *hrde-1* mutant pachytene nuclei showing the nuage marker GFP::PRG-1 (green) and *mex-6* RNA (magenta) under control or *mex-6* RNAi in the P0 generation. Scale bar is 2.5 µm. Images are representative of 3 worms examined for each condition. Results were consistent across three independent FISH experiments. **d**) Graph comparing the mean *mex-6* RNA FISH signal from the pachytene rachis in wild-type and *hrde-1* mutant F1 progeny of P0s administered either control (red) or *mex-6* (blue) RNAi. Each dot represents a single worm (n = 5 worms). Central black dot and error bars represent the mean and standard deviation, respectively. Values (arbitrary units) were normalized to *puf-5* RNA FISH signals visualized in same nuclei (Methods). WT values are the same as shown in Fig. S3A. Source data

HRDE-1 (also known as WAGO-9) is a germline-specific nuclear Argonaute required for inheritance of the RNAi-induced silenced state^12–14^. A normal RNAi response, including rapid loss of *mex-6* RNA in the cytoplasm and accumulation in nuage, was observed in the oocytes of *hrde-1* mutant P_0_ hermaphrodites (Fig. 4a and Extended Data Fig. 4a). However, the *hrde-1* mutants showed no change in the intensity distribution of nuclear puncta in the pachytene region (Fig. 4a–c). To explore the possibility that *hrde-1* mutants do not silence the *mex-6* locus, we examined the accumulation of *mex-6* transcripts in the rachis, the shared cytoplasm adjacent to pachytene nuclei. At 24 h, the levels of *mex-6* mRNA in the rachis declined by >90% in the wild-type worms compared with only about 50% in the *hrde-1* mutants (Fig. 4d,e). These observations suggest that *hrde-1* mutants fail to interfere with the production of *mex-6* transcripts in P_0_ hermaphrodites. We obtained similar results in a strain mutated for another component of the nuclear RNAi machinery, *nrde-2* (Extended Data Fig. 4b).

Failure to silence the *mex-6* locus was also observed in *hrde-1* F_1_ progeny. The intensities of nuclear puncta were similar in the *hrde-1* F_1_ *mex-6* RNAi and F_1_ control RNAi animals (Fig. 4a,c). As in the wild type, however, *hrde-1* F_1_ progeny accumulated *mex-6* transcripts in nuage in the pachytene region (Extended Data Fig. 4c). The levels of *mex-6* RNA in the pachytene rachis were higher in the *hrde-1* F_1_ than wild-type F_1_ animals from the *mex-6* RNAi condition but averaged only 50% of that observed in the *hrde-1* F_1_ control condition (Extended Data Fig. 4d). We conclude that *hrde-1* is required for silencing of the locus in P_0_ and F_1_ animals (nuclear response) but is not essential for RNA degradation in the cytoplasm and accumulation in nuage in P_0_ and F_1_ animals (cytoplasmic response).

### Accumulation in nuage requires *znfx-1* in P_0_ and F_1_ animals

**Fig. 5:**
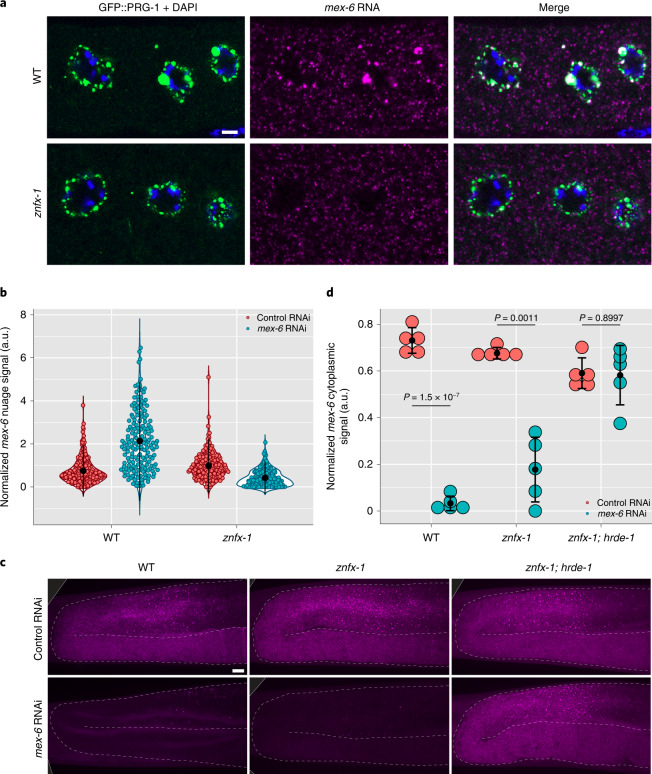
Enrichment of RNAi-targeted transcripts in nuage requires *znfx-1*. **a**, Single *z*-plane photomicrographs of oocytes in wild-type and *znfx-1* mutant P_0_ animals showing staining for the nuage marker GFP::PRG-1 (green), DNA (stained with DAPI; blue) and *mex-6* RNA (magenta) after 4 h of RNAi treatment. Scale bar, 2.5 µm. Images are representative of five worms examined for each condition. **b**, Comparison of the mean *mex-6* RNA FISH signal in oocyte nuage in wild-type and *znfx-1* mutant P_0_ animals after 4 h of RNAi treatment. Each dot represents an individual nuage granule. Nuage granules were quantified across five worms (the exact number of nuage granules quantified for each condition are provided in Source Data). **c**, Maximum projection photomicrographs of germlines showing *mex-6* RNA (magenta) in F_1_ wild-type, *znfx-1* mutant and *znfx-1;* *hrde-1-*double-mutant animals derived from P_0_ animals exposed to the indicated RNAi treatment. Scale bar, 10 µm. Images are representative of eight worms examined for each condition. **a**,**c**, Results were consistent across three independent FISH experiments. **d**, Comparison of the mean cytoplasmic *mex-6* RNA FISH signal in the pachytene region of *znfx-1* and *znfx-1;* *hrde-1* mutant F_1_ progeny derived from animals exposed to the indicated RNAi treatment. Each dot represents one animal (*n* = 5 worms). *P* values were calculated using an unpaired two-tailed Student’s *t*-test. **b**,**d**, The central black dot and error bars represent the mean and s.d., respectively. Values (arbitrary units, a.u.) were normalized to *puf-5* RNA FISH signals visualized in the same nuage granules (**b**) or germlines (**d**; Methods). Source data

**Extended Data Fig. 5:**
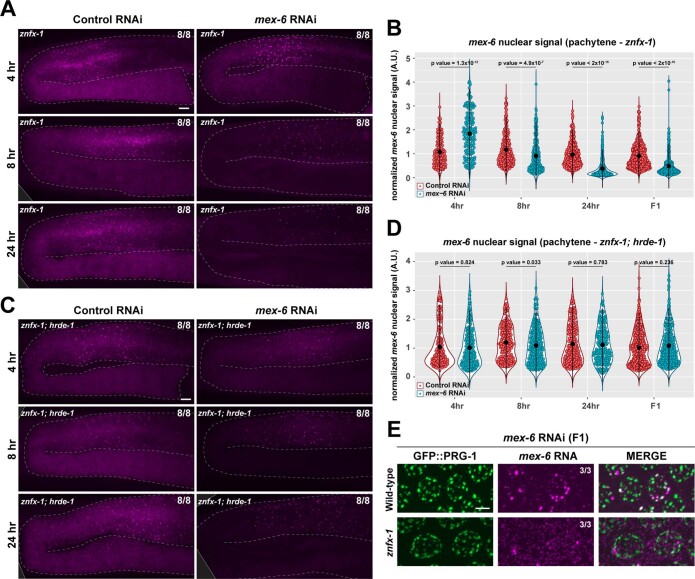
*mex-6* RNA patterning in *znfx-1* and *znfx-1; hrde-1* mutants upon RNAi. **a**) Maximum projection photomicrographs of *znfx-1* P0 mutant germlines showing *mex-6* RNA at the indicated time points following either control or *mex-6* RNAi. Scale bar is 10 µm. Images are representative of 8 wild-type worms examined for each condition. Results were consistent across three independent FISH experiments. **b**) Graph comparing the maximum *mex-6* nuclear FISH signal in control (red) vs *mex-6* (blue) RNAi at the indicated time points following RNAi in *znfx-1* P0 animals. Each dot of the violin plot represents one nucleus Nuclei were quantified across 3 worms (see Extended Data Fig. 5 source data for the exact number of nuclei quantified for each condition). Values (arbitrary units) were normalized to *puf-5* RNA FISH signals visualized in same nuclei (Methods). Central black dot and error bars represent the mean and standard deviation, respectively. P values were calculated using an unpaired two-tailed Wilcoxon rank-sum test. Refer to Fig. 2d for a comparison to wildtype. **c**) Maximum projection photomicrographs of *znfx-1; hrde-1* P0 germlines showing *mex-6* RNA at the indicated time points following either control or *mex-6* RNAi. Scale bar is 10 µm. Images are representative of 8 wild-type worms examined for each condition. Results were consistent across two independent FISH experiments. **d**) Graph comparing the maximum *mex-6* nuclear FISH signal in control (red) vs *mex-6* (blue) RNAi at the indicated time points following RNAi in *znfx-1; hrde-1* P0 animals. Each dot of the violin plot represents one nucleus Nuclei were quantified across 3 worms (see Extended Data Fig. 5 source data for the exact number of nuclei quantified for each condition). Values (arbitrary units) were normalized to *puf-5* RNA FISH signals visualized in same nuclei (Methods). Central black dot and error bars represent the mean and standard deviation, respectively. P values were calculated using an unpaired two-tailed Wilcoxon rank-sum test. **e**) Maximum projection photomicrographs of F1 wild-type and *znfx-1* mutant pachytene nuclei showing the nuage marker GFP::PRG-1 (green) and *mex-6* RNA (magenta) following administration of *mex-6* RNAi in the P0 generation. Scale bar is 2.5 µm. Images are representative of 3 wild-type worms examined for each condition. Results were consistent across three independent FISH experiments. Source data

ZNFX-1 is an SF1 helicase domain-containing zinc finger protein that, like HRDE-1, is required for inheritance of the RNAi-induced silenced state^21,22^. Unlike HRDE-1, which is primarily nuclear, ZNFX-1 localizes to nuage (Z granules)^22^. We found that the *mex-6* cytoplasmic transcripts in *znfx-1* P_0_ animals were rapidly degraded upon *mex-6* RNAi as in the wild type (Extended Data Fig. 5a). However, *mex-6* transcripts failed to accumulate in nuage (Fig. 5a,b).

We detected an increase in the intensity distribution of *mex-6* pachytene nuclear puncta in *znfx-1* mutants at the 4 h time point, 4 h earlier than the wild-type group (Extended Data Fig. 5a,b). This premature peak was followed by a decrease to levels lower than the non-RNAi condition by 24 h (Extended Data Fig. 5a,b). No changes in nuclear signal were observed in the *znfx-1;* *hrde-1* double-mutant animals, indicating that the nuclear response in the *znfx-1* mutants was dependent on *hrde-1*, as in the wild-type animals (Extended Data Fig. 5c,d). We conclude that *znfx-1* is required for robust recruitment of *mex-6* transcripts to nuage but not for RNA degradation in the cytoplasm or for engagement of the nuclear RNAi machinery in P_0_ animals. Despite a failure to silence the *mex-6* locus and enrich *mex-6* transcripts in nuage, *znfx-1;* *hrde-1* P_0_ animals still showed a rapid loss of cytoplasmic *mex-6* RNA throughout the germline, confirming that neither ZNFX-1 nor HRDE-1 is required for RNA turnover in the cytoplasm of P_0_ animals (Extended Data Fig. 5c).

In *znfx-1* F_1_ animals derived from *mex-6* RNAi fed P_0_s, we observed a partial reduction (approximately 50%) in cytoplasmic accumulation of *mex-6* transcripts in the pachytene rachis and no accumulation in nuage in the pachytene region (Fig. 5c,d and Extended Data Fig. 5e). The intensity distribution of nuclear puncta was reduced just as it was observed for the wild-type F_1_ group (Extended Data Fig. 5b). This reduction was dependent on *hrde-1*, as the nuclear puncta intensities of the *znfx-1;* *hrde-1* F_1_ animals matched that of the *znfx-1*; *hrde-1* F_1_ control RNAi animals (Extended Data Fig. 5c,d). We conclude that *znfx-1* is not required for silencing of the locus in P_0_ and F_1_ animals (nuclear response) but is required for the accumulation of targeted transcripts in nuage in P_0_ and F_1_ animals (cytoplasmic response).

### *hrde-1* and *znfx-1* contribute additively to silencing in F_1_ animals

**Extended Data Fig. 6:**
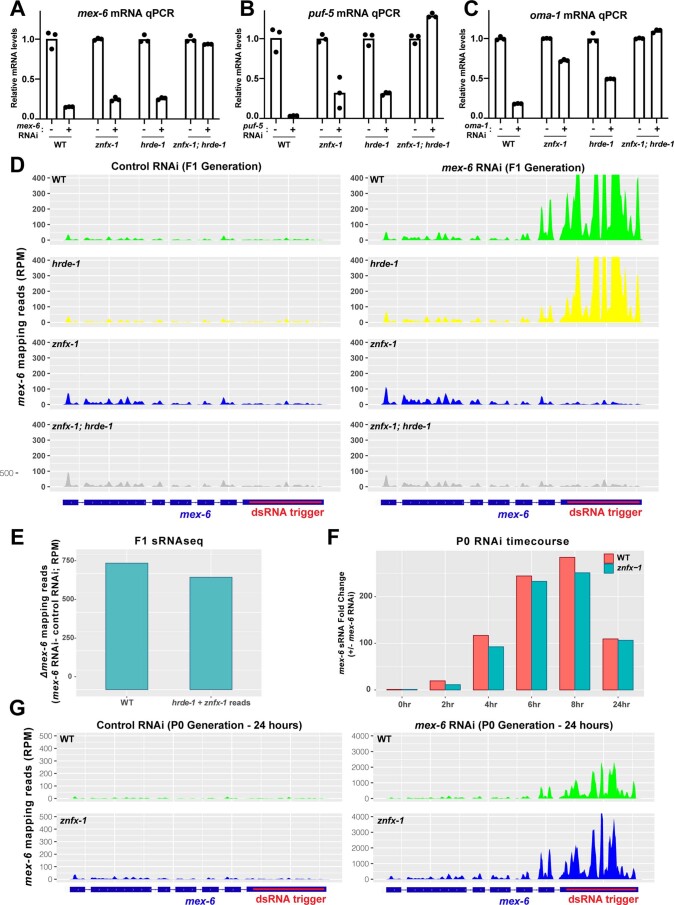
RNAi inheritance is dependent upon both *znfx-1* and *hrde-1*. **a-c**) Graphs showing relative mRNA levels comparing control (-; set to 1) and RNAi conditions (+) in P0 worms of the indicated genotypes. RT-qPCR Ct values in each sample were normalized to *tbb-2* RT-qPCR Ct values in the same sample and to the control RNAi condition (see Methods). Points indicate three technical replicates for each condition from a single source of biological RNA. **d**) Genome browser view of sRNA seq reads mapping to the *mex-6* locus in the genotypes and RNAi conditions indicated. Same data as Fig. 6b but values for the control and *mex-6* RNAi conditions are shown separately. Reads were averaged between two technical sequencing replicates from a single source of biological RNA for each genotype/condition. **e**) Graph comparing *mex-6* sRNA reads induced by RNAi in WT compared to the sum of the sRNA reads induced by RNAi in the *hrde-1* and *znfx-1* single mutants. See Methods for calculations. Reads were averaged between two technical sequencing replicates from a single source of biological RNA for each genotype. **f**) Graph showing the fold increase in sRNAseq reads mapping to the *mex-6* transcript in wild-type and *znfx-1* P0s at the indicated time points following either control (red) or *mex-6* (blue) RNAi. Each bar represents the *mex-6* RPM of a single biological replicate. **g**) sRNAs mapping to the *mex-6* locus in the P0 generation in WT and *znfx-1* mutants under control and *mex-6* RNAi conditions. Unlike F1 *znfx-1* animals (Fig. S6D), P0 *znfx-1* animals accumulate sRNAs in the trigger region. Each genome browser view is derived from sequencing of a single biological replicate. Source data

Unlike in P_0_ animals, the cytoplasmic *mex-6* RNA levels in *znfx-1;* *hrde-1* F_1_ animals were indistinguishable from the control condition, indicating that *znfx-1* and *hrde-1* are both required for maximal silencing in F_1_ animals (Fig. 5c,d). To examine this further, we compared the *mex-6* RNA levels in the wild-type as well as *znfx-1*, *hrde-1* and *znfx-1;* *hrde-1* mutant F_1_ animals using quantitative PCR with reverse transcription (Extended Data Fig. 6a). These experiments confirmed partial silencing of *mex-6* transcripts in the single mutants and complete loss of silencing in the F_1_ double mutants (Extended Data Fig. 6a). We obtained similar results when we targeted two other germline-expressed genes by RNAi (*oma-1* and *puf-5*; Extended Data Fig. 6b,c). We conclude that *hrde-1* and *znfx-1* contribute independently to silencing in F_1_ worms and are required additively for maximal silencing.

### *hrde-1* and *znfx-1* are responsible for distinct sRNA populations

**Fig. 6:**
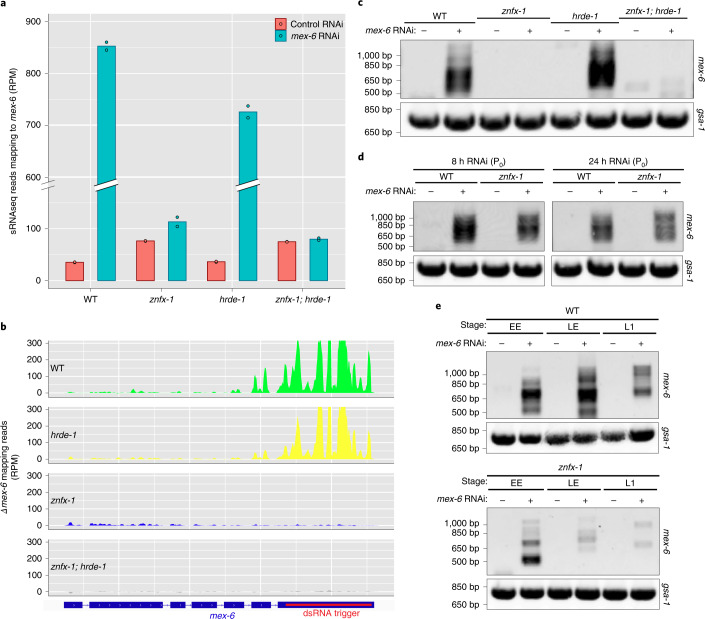
ZNFX-1 and HRDE-1 function in separate pathways contributing to RNAi inheritance. **a**, Number of sRNAseq reads (normalized per million, RPM) mapping to the *mex-6* transcript in wild-type as well as *znfx-1*, *hrde-1* and *znfx-1;* *hrde-1* mutant F_1_ progeny derived from animals exposed to the indicated RNAi treatment. This experiment was performed once and each dot represents a technical replicate. **b**, Genome browser view of sRNAseq reads mapping to the *mex-6* locus induced by *mex-6* RNAi in wild-type as well as *hrde-1*, *znfx-1* and *znfx-1;* *hrde-1* mutant F_1_ progeny. The sRNAseq reads were binned across the *mex-6* gene and the number of reads in each bin under the control RNAi condition were subtracted from the number of reads in each respective bin under the *mex-6* RNAi condition. See Extended Data Fig. 6d for raw reads shown separately for each condition. **c**, Gel showing PCR amplification of pUGylated *mex-6* RNA from lysates derived from the F_1_ progeny of animals with the indicated genotype treated with control or *mex-6* RNAi (top). **d**, Gel showing PCR amplification of pUGylated *mex-6* RNA from lysates derived from P_0_ animals with the indicated genotype and treated for 8 (left) or 24 h (right) with control or *mex-6* RNAi (top). **e**, Gel showing PCR amplification of pUGylated *mex-6* RNA from lysates derived from early embryos (EE), late embryos (LE) and first larval stage (L1) F_1_ progeny of animals with the indicated genotype treated with control or *mex-6* RNAi. **c**–**e**, The *gsa-1* transcript has a genomically encoded 18-nucleotide poly(UG) stretch and is used here as a positive control for pUG amplification (bottom; Shukla et al.^9^). Gels are representative of two independent experiments; −, control RNAi; +, *mex-6* RNAi. Source data

The additive phenotype of the *znfx-1;* *hrde-1* double mutant suggested that *hrde-1* and *znfx-1* function in separate mechanisms to maintain nuclear and cytoplasmic silenced states. To examine this possibility, we sequenced sRNAs in *hrde-1*, *znfx-1* and *znfx-1;* *hrde-1* mutant as well as wild-type F_1_ animals. As expected, the wild-type F_1_ worms exhibited a 23-fold increase in sRNAs mapping to the *mex-6* locus compared with the control F_1_ animals, with a dominant peak corresponding to the location targeted by the dsRNA trigger (Fig. 6a,b). We also detected an increase in sRNAs at the *mex-6* locus in the *hrde-1* and *znfx-1* mutant F_1_ animals, but to different extents. The increase in sRNAs reached 83% of the wild type in the *hrde-1* mutants but only 6% of the wild type in the *znfx-1* mutants (Fig. 6a,b). Wan et al. also reported low levels of sRNAs in *znfx-1* mutant F_1_ animals^22^. The sRNAs accumulated preferentially in the trigger region in *hrde-1* mutants but not in *znfx-1* mutants (Fig. 6b and Extended Data Fig. 6d). The sRNAs distributed throughout the *mex-6* locus, with a slight bias for the 5′ end, in the *znfx-1* mutants. Consistent with the complete lack of inherited RNAi response, the *znfx-1;* *hrde-1* double mutants exhibited no significant differences in sRNAs in *mex-6* compared with control F_1_ animals (Fig. 6a,b). Together, these observations suggest that *hrde-1* and *znfx-1* are required for the amplification of distinct pools of sRNAs across the *mex-6* locus, with *znfx-1* required for the bulk of sRNA generation, especially around the sequence targeted by the original trigger. We noticed that the number of sRNA reads mapping to the *mex-6* locus in *znfx-1* and *hrde-1* single mutants added up to 89% of the reads observed in wild-type F_1_ animals (Extended Data Fig. 6e; see Methods). This observation confirms that the ZNFX-1 and HRDE-1 amplification cycles function mostly independently, with possibly some synergy between the two cycles accounting for approximately 10% of sRNAs observed in the wild types.

To determine whether *znfx-1* is also required for sRNA amplification in P_0_ animals, we sequenced sRNAs in wild-type and *znfx-1* mutant hermaphrodites at different time points following feeding onset. We observed an increase of approximately 200-fold in sRNA accumulation at the *mex-6* locus in the wild-type and *znfx-1* P_0_ animals compared with control conditions (Extended Data Fig. 6f,g). The increase in sRNAs was slightly lower in the *znfx-1* mutants compared with the wild types (reduction of approximately 16%), suggesting that *znfx-1*, although not essential, contributes modestly to sRNA amplification in P_0_ animals. Unlike in the F_1_ generation, *znfx-1* P_0_ animals accumulated sRNAs predominantly in the trigger region, as observed in the wild type (Extended Data Fig. 6g). These observations indicate that *znfx-1* is not essential for sRNA amplification in P_0_ animals exposed to the trigger but is required in the F_1_ generation (Fig. 6a,b).

### *znfx-1* is required to sustain pUGylation in F_1_ progeny

**Extended Data Fig. 7:**
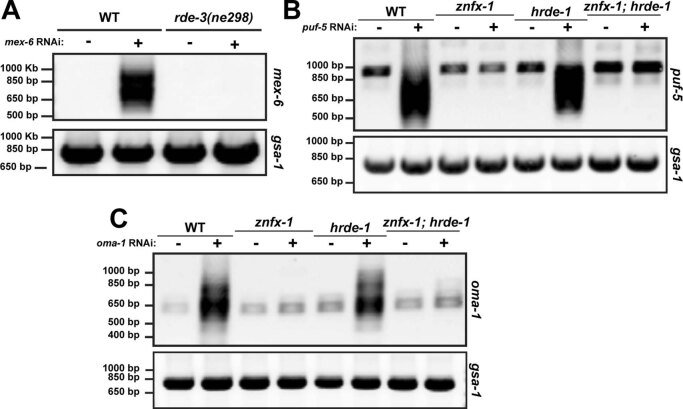
pUGylation in the F1 generation is dependent upon ZNFX-1, but not HRDE-1 following RNAi. **a**) Gel showing PCR amplification of pUGylated *mex-6* RNAs from lysates derived from WT and *rde-3(ne298)* P0 animals treated with control (‘−‘) or *mex-6* (‘+’) RNAi (top panel). *rde-3* mutants lack the pUGylase and do not produce pUGylated transcripts. *gsa-1* is the pUG amplification control (bottom panels). **b-c**) Gels showing PCR amplification of *puf-5* or *oma-1* pUGylated RNAs from lysates derived from P0 animals of the indicated genotypes treated with control (‘-“) or experimental (‘+’) RNAi as indicated. Red dots indicate position of non-specific bands. The pUGylation experiments in a-c have been performed once. Source data

A minority of sRNAs mapping to the trigger region correspond to primary sRNAs, with the majority corresponding to secondary sRNAs templated from pUGylated transcripts^9,29^. To determine whether *hrde-1* or *znfx-1* are required for pUGylation, we amplified pUGylated *mex-6* transcripts from wild-type and mutant F_1_ animals (position of primers shown in Extended Data Fig. 2a). As expected, we detected (see *mex-6* pUGylated transcripts) in wild-type F_1_ animals from *mex-6* RNAi fed P_0_s, but not in the F_1_ controls (Fig. 6c) or in worms mutated for the pUGylase RDE-3 (ref. ^9^; Extended Data Fig. 7a). We detected pUGylated *mex-6* transcripts in *hrde-1* mutant F_1_ adults but not in *znfx-1* or *znfx-1;* *hrde-1* F1 mutant adults (Fig. 6c). Similar results were obtained in experiments with *puf-5* and *oma-1* mutants (Extended Data Fig. 7b,c). In contrast to F_1_ adults, we detected pUGylated transcripts in *znfx-1* P_0_ adults (Fig. 6d) and F_1_ embryos (Fig. 6e). We conclude that *znfx-1* is not required for the initial production of pUGylated transcripts in the P_0_ generation but is required to sustain production and/or maintenance in adult F_1_ animals.

### ZNFX-1 associates with pUGylated transcripts

**Fig. 7:**
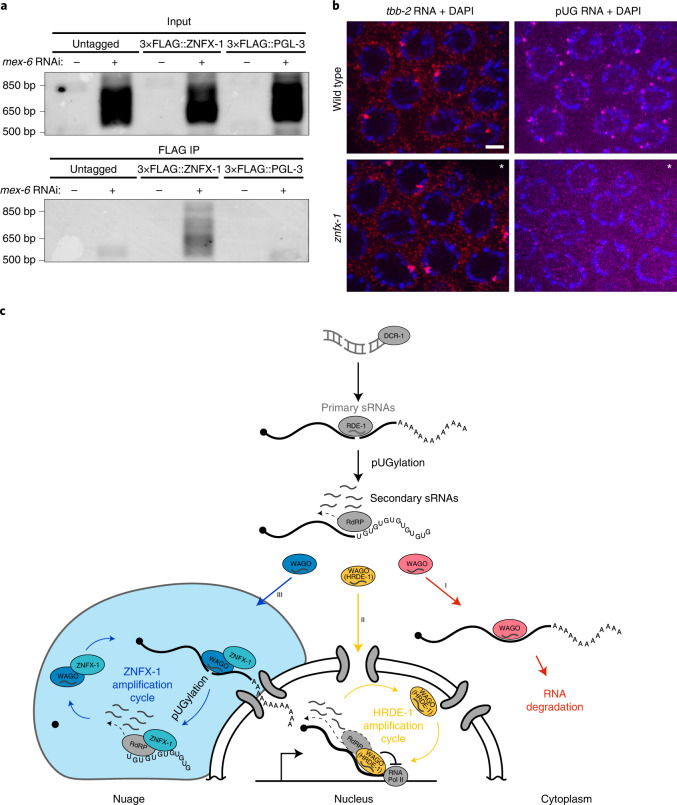
ZNFX-1 immunoprecipitates with pUGylated RNAs and is required for localization of pUGylated RNAs to nuage. **a**, Gel showing amplification of pUGylated *mex-6* RNA from input or FLAG immunoprecipitates (IP) of lysates collected from adult worms grown for 8 h on either *mex-6* (+) or *puf-5* (−) RNAi. PGL-3 is a nuage protein that serves here as a negative control. Refer to Extended Data Fig. 8a for western blots demonstrating efficient immunoprecipitation of 3×FLAG-tagged ZNFX-1 and PGL-3. Gels are representative of three independent pull downs. **b**, Photomicrographs of pachytene nuclei in dissected germlines from wild-type (top) and *znfx-1* mutant (bottom) animals showing staining for endogenous pUGylated RNAs (magenta), control *tbb-2* RNA (red) and DNA (stained with DAPI; blue). The contrast of the *znfx-1* images labelled with an asterisk were adjusted to match the intensity of the *tbb-2* signal in the wild type. See Extended Data Fig. 8c for unadjusted photomicrographs. Scale bar, 2.5 µm. Images are representative of six worms examined for each condition. Results were consistent across three independent FISH experiments. **c**, Working model for exogenous RNAi. The dsRNA trigger is processed into primary sRNAs that load with RDE-1 to target complementary transcripts for pUGylation. The pUGylated transcripts recruit RdRPs to generate secondary sRNAs in the cytoplasm (grey). Secondary sRNAs bind to secondary Argonautes (WAGO proteins), which initiate three distinct pathways. The first pathway (red) leads to degradation of cytoplasmic transcripts with no further sRNA amplification. On its own, this pathway is sufficient to silence gene expression in P_0_ animals exposed to dsRNA triggers but is insufficient to propagate the RNAi response across generations. A second pathway (yellow), dependent on the nuclear Argonaute HRDE-1, partially silences the locus and uses nascent transcript as templates for the production of tertiary sRNAs. A third pathway (blue), dependent on the nuage helicase ZNFX-1, enriches targeted transcripts in nuage, where they are pUGylated and used as templates for further tertiary sRNA amplification. Tertiary sRNAs feed back into their respective cycles, ensuring inheritance of the silenced state. The *hrde-1* and *znfx-1* sRNA amplification pathways are not essential for silencing in P_0_ animals but are required additively for full silencing in F_1_ animals. Possible crosstalk between the HRDE-1 and ZNFX-1 cycles is not shown. See Discussion for further considerations. Source data

**Extended Data Fig. 8:**
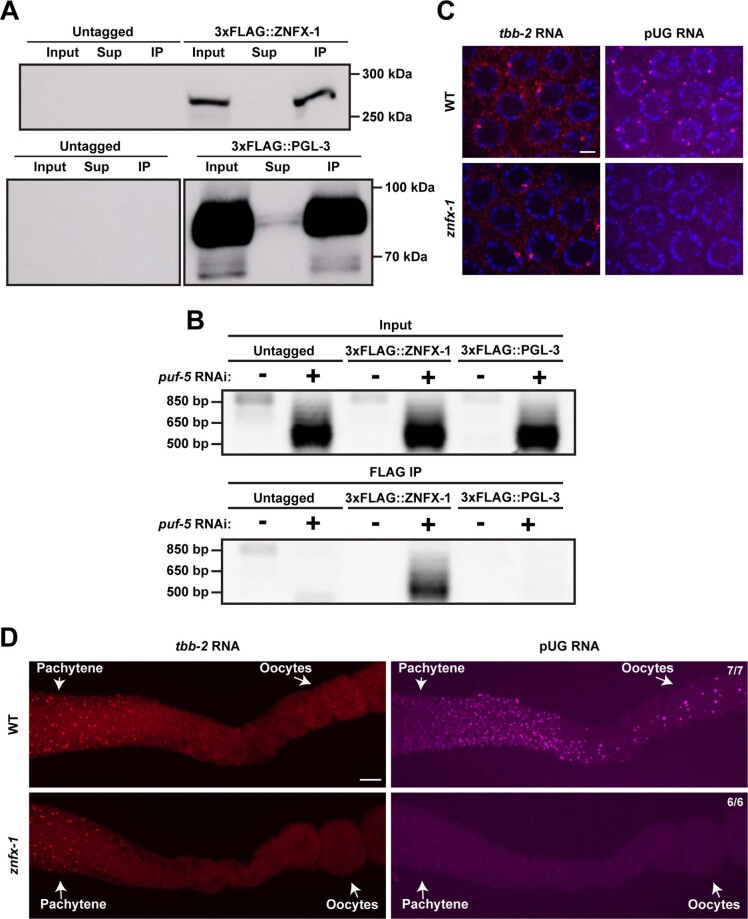
ZNFX-1 interacts/colocalizes with pUGylated transcripts. **a**) Anti-FLAG western blots to control for the efficiency of immunoprecipitation experiments shown in Fig. 7. ‘Input’ represents 1% of the input sample prior to immunoprecipitation; ‘Sup’ represents 1% of the supernatant following immunoprecipitation; ‘IP’ represents 1% of the immunoprecipitation sample following FLAG elution. Blots are representative of three independent pull downs. **b**) Gel showing PCR amplification of pUGylated *puf-5* RNA from input (top panel) or FLAG immunoprecipitates (bottom panel) from animals where the *znfx-1* or *pgl-3* locus is untagged or tagged with 3xFLAG. Lysates were collected from adult worms grown for 8 h on either *puf-5* (‘+’) or *mex-6* (‘-’) RNAi and are the same lysates used in Fig. 7a. Gels are representative of three independent pull downs. **c**) Same images as in Fig. 7b without contrast adjustment. See Fig. 7b for further details. **d**) Maximum projection photomicrographs of dissected wild-type and *znfx-1* mutant germlines showing pUG RNA FISH (magenta) and control *tbb-2* RNA. The pachytene region and oocytes are indicated with arrows. In this example, the dissected germline is extended rather than bent as shown in Fig. 1. Scale bar is 10 µm. Images are representative of 7 wild-type worms examined and 6 *znfx-1* worms examined. Results were consistent across three independent FISH experiments. Source data

ZNFX-1 immunoprecipitates with transcripts targeted by RNAi^22^. To determine whether ZNFX-1 interacts with pUGylated transcripts, we immunoprecipitated FLAG-tagged ZNFX-1 from animals exposed to RNAi triggers for 8 h and examined the immunoprecipitates for pUGylated RNAs^9,19^. As a control we also tested immunoprecipitates of FLAG-tagged PGL-3, a protein that like ZNFX-1 accumulates in nuage. We detected specific pUGylated transcripts in ZNFX-1 precipitates but not PGL-3 precipitates, despite the higher abundance of PGL-3 (Fig. 7a and Extended Data Fig. 8a,b). We conclude that ZNFX-1 exists in a complex with pUGylated RNAs.

Animals not exposed to exogenous triggers naturally contain pUGylated transcripts due to targeting by endogenous sRNAs^9^. Using a poly(AC) probe, we detected endogenous pUGylated transcripts in nuage in the pachytene region and in oocytes (Fig. 7b and Extended Data Fig. 8d) as reported previously^9^. Remarkably, in *znfx-1* mutant germlines, the poly(AC) signal was strongly reduced in pachytene and oocyte nuage (Fig. 7b and Extended Data Fig. 8d). We conclude that ZNFX-1 is required for the accumulation of endogenous pUGylated RNAs in nuage.

## Discussion

Together with previous studies, our findings suggest the following model for silencing by an exogenous dsRNA trigger (Fig. 7c). Primary sRNAs loaded on Argonaute RDE-1 recognize complementary transcripts and mark them for cleavage, pUGylation and synthesis of secondary sRNAs by RdRPs^9–11,27,29,30^. Secondary sRNAs load on HRDE-1 and other Argonautes^11–14,31^ to activate three parallel silencing pathways. In the first pathway (Pathway I; red in Fig. 7c), WAGO proteins tag transcripts in the cytoplasm for rapid degradation by an unknown mechanism. In the second pathway (II; yellow in Fig. 7c), HRDE-1 shuttles into the nucleus to initiate ‘nuclear RNAi’, a silencing programme that suppresses, but does not eliminate, productive transcription of the locus. In the third pathway (III; blue in Fig. 7c), WAGO proteins that associate with ZNFX-1 recruit a subset of targeted transcripts to nuage and initiate a new cycle of pUGylation and sRNA amplification. Only the HRDE-1 and ZNFX-1 cycles generate tertiary sRNAs that feed back into their respective cycles to generate parallel self-reinforcing sRNA amplification loops. The HRDE-1 and ZNFX-1 amplification loops are transmitted to the next generation independently of each other and both are required for maximum silencing in F_1_ progeny. In the following sections, we summarize evidence supporting the three silencing pathways and discuss remaining open questions.

### Pathway I: secondary sRNAs induce RNA degradation in the cytoplasm

Under our RNAi conditions, we detected a reduction in transcript levels in the cytoplasm after 4 h of feeding, eventually reaching 95% reduction by 24 h. As expected^27,28,32^, mRNA degradation was dependent on the primary Argonaute RDE-1 and MUT-16, a scaffolding protein required for amplification of secondary sRNAs. Messenger RNAs targeted by microRNAs for degradation have been reported to enrich in P bodies^33,34^, cytoplasmic RNA granules that enrich components of the RNA degradation machinery^35^. In our feeding experiments, we observed enrichment of targeted mRNAs in nuage condensates, but this enrichment was not linked to RNA degradation. Most strikingly, no nuage enrichment was observed in *znfx-1* mutants, despite normal RNA degradation in these animals. We conclude that, unlike microRNA-induced RNA degradation, RNAi-induced RNA degradation does not require visible RNA enrichment in cytoplasmic granules. The robust sRNA amplification observed in *znfx-1* mutant P_0_ animals also suggests that secondary sRNA amplification initiated by primary sRNAs occurs in bulk cytoplasm or, at a minimum, does not require accumulation of targeted transcripts in granules. We cannot exclude that transit through nuage or some other RNA granules, in the absence of visible accumulation, is required for secondary sRNA amplification and/or RNA degradation. The RDE-1-initiated cycle of pUGylation and sRNA amplification is sufficient to eliminate most cytoplasmic transcripts in animals exposed to the dsRNA trigger. However, this cycle is not self-perpetuating and on its own eventually self-extinguishes, leaving no memory of the RNAi response.

### Pathway II: HRDE-1 reduces but does not eliminate transcription

We detected a transient increase in nascent transcripts after 6 h of RNAi in P_0_ animals. This response requires the nuclear Argonaute HRDE-1 and may reflect stalling of RNA polymerase II and/or pre-mRNA processing, causing nascent transcripts to accumulate at the locus. Stalling of RNA polymerase has previously been implicated in nuclear RNAi^36,37^ and several lines of evidence have linked RNAi and splicing, including apparent co-evolution of the RNAi and splicing machineries^38^, splicing factors identified as HRDE-1 interactors^39,40^, sRNA defects associated with mutations or knock down of spliceosome components^41,42^ and insensitivity to nuclear RNAi of an endogenous transcript whose introns were removed by genome editing^43^.

At 24 h post feeding and even more acutely in F_1_ animals, we observed a decrease in nascent transcripts, which may reflect a reduction in transcription initiation at the locus. The nuclear RNAi machinery deposits chromatin marks at the locus predicted to decrease transcription^44–47^. Despite this apparent decrease in transcriptional output, we continued to observe transcripts in perinuclear nuage even in F_1_ animals, indicating that a baseline level of transcription and export is maintained at the silenced locus. In *S. pombe*, transcription is maintained at the silent locus but export is blocked and replaced by rapid degradation of nuclear transcripts^8^. This difference may reflect a *C. elegans*-specific adaptation that allows mature transcripts to be used as templates for sRNA amplification in perinuclear condensates (see Pathway III).

The HRDE-1 cycle generates sRNAs that map throughout the locus without preference for the trigger area and with a slight preference for the 5′ end of the transcript. A similar pattern was described previously in the context of transgenes and endogenous transcripts targeted by endogenous sRNA pathways, and was found to be dependent on the nuclear RNAi machinery^15^. One hypothesis is that the 5′ bias is due to RdRPs that use nascent transcripts as templates for sRNA synthesis as described in *S. pombe*. Consistent with this hypothesis, the nuclear RNAi machinery has been shown to interact with pre-mRNAs at the locus, which naturally exhibit a 5′ bias^36,48^. We suggest that HRDE-1, initially loaded with secondary sRNAs templated in the cytoplasm, initiates a nuclear cycle of sRNA amplification by recruiting an RdRP to nascent transcripts. The HRDE-1 cycle generates tertiary sRNAs, which in turn become complexed with HRDE-1 to perpetuate the cycle. The RdRP EGO-1 has been reported to localize in nuclei^49^ but a specific molecular interaction between EGO-1 and HRDE-1 has not been reported. However, analyses of silencing in operons have provided indirect evidence for an RdRP activity in nuclei^15,50–52^. Although we favour a model where HRDE-1 and associated machinery use nascent transcripts to direct sRNA synthesis (Fig. 7c, yellow), we cannot exclude the possibility that HRDE-1-dependent sRNA amplification occurs outside the nucleus after export into the cytoplasm. Investigation into the factors that support HRDE-1-dependent sRNA production is an important future goal.

### Pathway III: ZNFX-1 memorializes targeted RNAs in nuage

The HRDE-1 amplification cycle is insufficient for maximum silencing in F_1_ progeny. A second cycle dependent on the nuage protein ZNFX-1 is also required. The ZNFX-1 cycle generates sRNAs focused primarily on the area targeted by the original trigger and is responsible for the bulk of sRNA production in F_1_ animals. ZNFX-1 is required for the production and/or maintenance of pUGylated transcripts in F_1_ adults, for enrichment of RNAi-targeted transcripts and pUGylated RNAs in nuage, and can be immunoprecipitated with pUGylated transcripts. Together, these observations suggest that ZNFX-1 maintains a pool of silenced transcripts in nuage to enable their use as templates for sRNA amplification. Compartmentalization in nuage may serve to protect transcripts from RNA degradation enzymes in the cytoplasm and facilitate recognition by the pUGylase MUT-3 and RdRPs for synthesis of tertiary sRNAs (Fig. 7). Consistent with this model, ZNFX-1 has been reported to immunoprecipitate with the secondary Argonautes WAGO-1 and WAGO-4, and the RdRP EGO-1 (refs. ^21,22,53^). Presumably, tertiary sRNAs generated in the ZNFX-1 loop feed back into additional cycles of pUGylation and sRNA amplification to ensure propagation of sRNA amplification across generations. Because this self-perpetuating cycle is initiated by secondary sRNAs that target the trigger region, the ZNFX-1 cycle preferentially amplifies sRNAs near the trigger. ZNFX-1 has been proposed to help maintain uniform distribution of RdRPs on silenced transcripts, based on the observation that endogenous sRNAs exhibit a 5′ bias in *znfx-1* mutants^21^. We suggest another possible explanation: in *znfx-1* mutants, the only sRNAs remaining are those created by nuclear RNAi pathways, which are naturally 5′-biased given that they are templated from nascent transcripts.

It has been suggested that, in *C. elegans*, initiation of sRNA amplification by non-primary sRNA–Argonaute complexes is limited in vivo to prevent dangerous runaway loops^20^. We speculate that enrichment of ZNFX-1 in nuage places the ZNFX-1 amplification loop under tight regulation by competing sRNA pathways (for example, piRNAs) that protect transcripts from permanent silencing^19^.

A role for ZNFX-1 in promoting sRNA amplification is consistent with the role of Hrr1, the *S. pombe* orthologue of ZNFX-1, which functions with an RdRP^54^ and the predicted poly-A polymerase Cid12, which may be relevant to the role we propose here for ZNFX-1 in promoting the synthesis and/or stabilization of pUGylated transcripts. However, unlike ZNFX-1, Hrr1 is nuclear and targets nascent transcripts^54^. ZNFX-1 homologues in mice and humans function in the primary immune response against RNA viruses and bacteria^55–58^. Mammalian ZNFX1 recognizes viral RNAs and localizes to the surface of mitochondria^55^. Nuage-like compartments have been observed on the surface of mitochondria in several germ cell types, including mouse sperm^59^. A common function for ZNFX1 orthologues in higher eukaryotes may therefore be to recognize and isolate transcripts in perinuclear or perimitochondrial nuage-like compartments for long-term silencing.

### The HRDE-1 and ZNFX-1 sRNA amplification loops function in parallel

In contrast to RDE-1-initiated sRNA amplification, the HRDE-1 and ZNFX-1 programmes are self-sustaining cycles that maintain a pool of targeted transcripts for use as templates for sRNA amplification. In our RNAi feeding paradigm, the HRDE-1 and ZNFX-1 programmes were both required for full silencing in F_1_ animals. However, it is possible that reliance on the HRDE-1 or ZNFX-1 programmes will vary between loci and in response to other silencing triggers, such as endogenous sRNAs. Different genetic requirements for RNAi inheritance in different contexts have been previously documented^60,61^.

Although our analyses suggest that the HRDE-1 and ZNFX-1 pathways function primarily independently of each other, two lines of evidence hint at possible crosstalk. First, the sum of *mex-6* sRNAs induced by RNAi in *hrde-1* and *znfx-1* F_1_ animals added up to only 89% of what is observed in the wild type. Although this observation needs to be repeated in different contexts to ensure reproducibility, it suggests that sRNAs produced by one amplification cycle extend sRNA production in the other cycle. Second, the nuclear RNAi response was accelerated in *znfx-1* P_0_ animals compared with the wild type, raising the possibility that the two cycles compete for secondary sRNAs and/or RdRPs in the early stages of the RNAi response. Alternatively, ZNFX-1 may antagonize HRDE-1-initiated transcriptional silencing to ensure sufficient production of mature mRNAs for use in the ZNFX-1 cycle. More complex interplays involving Argonautes that participate in multiple sRNA amplification mechanisms are also possible. How the RDE-1, HRDE-1 and ZNFX-1 sRNA amplification mechanisms coordinate in cells and across generations will be an important focus for future investigations.

## Methods

### Strains and maintenance

Strains were cultured at 20 °C on OP50 bacteria plated on NNGM medium or NA22 bacteria plated on Enriched Peptone medium. The following strains were used in this study: N2 (JH1), *znfx-1(gg561) II* (YY996)^22^, *hrde-1(tm1200) III* (YY538)^13^, *znfx-1(gg561) II;* *hrde-1(tm1200) III* (JH4054; this study), *prg-1(ne4523[gfp::tev::flag::prg-1]) I* (WM527)^64^, *znfx-1(gg544[3xflag::gfp::znfx-1])* (YY916)^22^, *prg-1(ne4523) I;* *znfx-1(gg561) II* (JH4055; this study), *prg-1(ne4523) I;* *hrde-1(tm1200) III* (JH4056; this study), *prg-1(ne4523) I;* *znfx-1(gg561) II;* *hrde-1(tm1200) III* (JH4057; this study), *prg-1(ne4523) I;* *rde-1(ne219) V* (JH4058; this study)*, prg-1(ne4523) mut-16(pk710) I* (JH4059; this study), *znfx-1(ne4355[3Xflag::tev::znfx-1]) II* (JH 4159; *ne4355* allele outcrossed from WM514)^21^, *pgl-3(ax4516[pgl-3::3xFLAG]) V;* *meg-3(ax3054[meg-3::meGFP]) X* (JH4072; this study). The *pgl-3(ax4516)* allele was generated by clustered regularly interspaced short palindromic repeats–Cas9 genome editing^65^.

### RNA extraction and purification

Up to 100 µl of worms flash-frozen in liquid nitrogen and stored at −80 °C were resuspended in 1 ml TRIzol (Thermo Fisher, cat no. 15596026), subjected to three freeze–thaw cycles and incubated at room temperature for 5 min with shaking at 1,500 r.p.m. (Benchmark Scientific, model no. H5000-HC) and an additional 5 min without shaking. After the addition of 200 µl chloroform, the samples were shaken by hand for 15 s, followed by an incubation of 2–3 min at room temperature and 12,000*g* centrifugation at 4 °C for 15 min. The upper aqueous phase was removed and an equal volume of 95–100% ethanol was added. The samples were concentrated and purified using the Zymo RNA clean & concentrator kit columns (Zymo, cat no. R1017). On-column DNase I digestions with MgCl_2_ buffer (Thermo Fisher, cat no. EN0521) were used to remove contaminating DNA. The samples were eluted in water.

### Plasmid construction

RNAi plasmids were constructed using the L4440 vector and In-Fusion HD cloning kit (Takara Bio, cat no. 639650) transformed into Stellar competent cells (Takara Bio, cat no. 636766) and isolated using Qiagen mini-prep kits (cat no. 27104). Primers (Supplementary Table 1) were designed using the Takara Bio In-Fusion Cloning online design tool. NEB Phusion PCRs (NEB, cat no. M0531S) were conducted from reverse transcriptase reactions generated using a SuperScript VILO cDNA synthesis kit (Thermo Fisher, cat no. 11754050) and RNA was extracted from adult animals (see the ‘RNA extraction and purification’ section). The empty L4440 vector was used as a control RNAi construct.

### RNAi assays

We chose *mex-6* as a model transcript for our analysis because (1) *mex-6* is expressed in the pachytene region of the adult germline, where perinuclear condensates are prominent (Fig. 1); (2) *mex-6* is minimally targeted by endogenous sRNAs under non-RNAi conditions (Extended Data Fig. 1a) and (3) *mex-6* is a non-essential maternal-effect gene (redundant with *mex-5*) whose silencing does not affect germline development or morphology^66^.

RNAi constructs were transformed into HT115 bacteria and the transformants were cultured overnight in LB liquid medium containing 100 µg ml^−1^ ampicillin at 37 °C with vigorous shaking and used to inoculate a fresh LB–ampicillin culture (1:100 ratio; for example, 10 ml starter culture into 990 ml LB), cultured for 6.5 h with vigorous shaking at 37 °C and induced with isopropylthiogalactoside (IPTG; 500 µM total concentration) in the final 30 min. The cultures were spun down and resuspended in LB medium containing 100 µg ml^−1^ ampicillin and 500 µM IPTG (1/20 of the culture volume; for example, 50 ml for 1,000 ml of culture) and densely plated onto NNGM agar containing 100 µg ml^−1^ carbenicillin and 1 mM IPTG. The plates were allowed to dry before use.

Embryos were hatched in M9 overnight at 20 °C with shaking at 110 r.p.m. and plated onto NA22 bacteria cultured on Enriched Peptone medium. The adult worms were washed off the plates 60 h after plating, collected using a filter, re-plated onto either control (empty L4440 vector) or gene-specific RNAi plates for specified time periods and fixed (see the ‘FISH protocol’ section), used for RNA extraction (see RNA collection) or used to collect F_1_ embryos for RNAi inheritance assays.

For the RNAi inheritance assays, F_1_ embryos (isolated from P_0_ adults by bleaching) were synchronized by shaking overnight (110 r.p.m.) at 20 °C, plated onto NA22 plates for 72 h and collected for fixation, RNA extraction or to collect F_2_ embryos by bleaching. F_2_-synchronized animals in the first larval stage were plated for approximately 72 h onto NA22 plates before examination.

For experiments examining late and early F_1_ embryos, adult worms were fed RNAi for 24 h and bleached to isolate ‘early embryos’. Embryos laid on the plate were also collected and considered ‘late embryos’. Late embryo samples were also bleached to eliminate potential contamination by hatched animals in the first larval stage.

### FISH protocol

Stellaris Probe Designer (v4.2) was used to design smFISH probes (Supplementary Table 2), which were purchased with Quasar670 and Quasar570 dyes.

For FISH of the whole worm (undissected), 100 µl of live worms were fixed in 1,000 µl fixation buffer (1×PBS and 3.7% formaldehyde) on a rotating shaker at room temperature for 45 min, spun down at 3,000*g* in a table-top centrifuge and washed twice with 1,000 µl 1×PBS. The worms were pelleted and stored at 4 °C for at least 4 h in 1,000 µl of 75% ethanol. The samples were washed once with 1,000 µl of freshly prepared Stellaris Buffer A Mixture (10% deionized formamide, 20% Stellaris RNA FISH Wash Buffer A (Biosearch Technologies, cat no. SMF-WA1-60) and 70% RNase-free water) and resuspended in 100 µl of freshly prepared Hybe Buffer Mixture (for two-colour in situ hybridization, 85.5 µl Stellaris RNA FISH Hybridization Buffer (Biosearch Technologies; cat no. SMF-HB1–10), 9.5 µl deionized formamide, 2.5 µl of 5 µM probe 1 suspended in TE and 2.5 µl of 5 µM probe 2 suspended in TE) before overnight incubation at 37 °C. After the addition of 1,000 µl of freshly prepared Stellaris Buffer A Mixture at 37 °C for 30 min, the samples were resuspended in 1,000 µl Stellaris Buffer A Mixture with 5 ng ml^−1^ DAPI at 37 °C for 30 min, resuspended in Stellaris RNA FISH Wash Buffer B (Biosearch Technologies, cat no. SMF-WB1-20) for 5 min at room temperature and finally resuspended in Vectashield Antifade Mounting Medium with DAPI (VWR, cat no. H-1200-10) before placing on slides for microscopy.

For FISH of dissected germlines, worms in M9 with 10 mM levamisole were dissected to release germlines, freeze-cracked on dry ice, placed into cold (−20 °C) methanol, washed three times in PBS + 0.1% Tween 20 and fixed in 4% paraformaldehyde for 1 h at room temperature. The samples were washed on slides with Stellaris Buffer A Mixture, which was replaced with 100 µl of freshly prepared Hybe Buffer Mixture and incubated at 37 °C overnight. The slides were washed in 500 µl of freshly prepared Stellaris Buffer A Mixture, incubated in the same for 30 min at 37 °C, washed in 500 µl Stellaris Buffer A Mixture containing 5 ng ml^−1^ DAPI, incubated in the same for 30 min at 37 °C, washed with 500 µl Stellaris RNA FISH Wash Buffer B (Biosearch Technologies, cat no. SMF-WB1-20) and incubated in the same for 5 min at room temperature before replacing the buffer with Vectashield and sealing under a coverslip.

### RNAseq

For sRNAseq, 5 µg of total RNA was treated with 5′ polyphosphatase (20 U µg^−1^ RNA) for 30 min at 37 °C and purified using Zymo RNA clean & concentrator kit columns (Zymo, cat no. R1017). The treated RNA (1 µg) was inputted into an Illumina TruSeq small RNA library preparation kit (cat no. RS-200-0012) with 11 cycles of PCR amplification. The libraries were run on either a 6% Novex TBE gel or a 5% Criterion TBE gel and size-selected according to the Illumina protocol. Purified samples were sequenced on an Illumina HiSeq2500 system at the Johns Hopkins University School of Medicine Genetic Resources Core Facility.

For mRNAseq, 1 µg of total RNA isolated using TruSeq stranded total RNA library prep gold (Illumina, cat no. 20020598) was mixed with 2 µl of a 1:100 dilution of the ERCC RNA spike-in mix 1 (Thermo Fisher, cat no. 4456740). TruSeq RNA UD indexes were used for indexing (Illumina, cat no. 20022371) and libraries were pooled for sequencing on a NovaSeq6000 system at the Johns Hopkins University School of Medicine Genetic Resources Core Facility.

### High-throughput sequencing analyses

For the sRNAseq libraries, 5′ Illumina adaptor sequences were removed using the default settings of Cutadapt^67^ and reads that were longer than 30 nucleotides or shorter than 18 nucleotides were discarded. The libraries were aligned to the UCSC ce10 reference genome using HISAT2 (ref. ^68^). For assessing the number of reads mapping to the *mex-6* gene, the total number of reads aligning to *mex-6* were counted for two technical replicates and normalized to the number of singly aligned reads mapping to the genome (that is, library size) per million reads (RPM).

**Extended Data Fig. 9:**
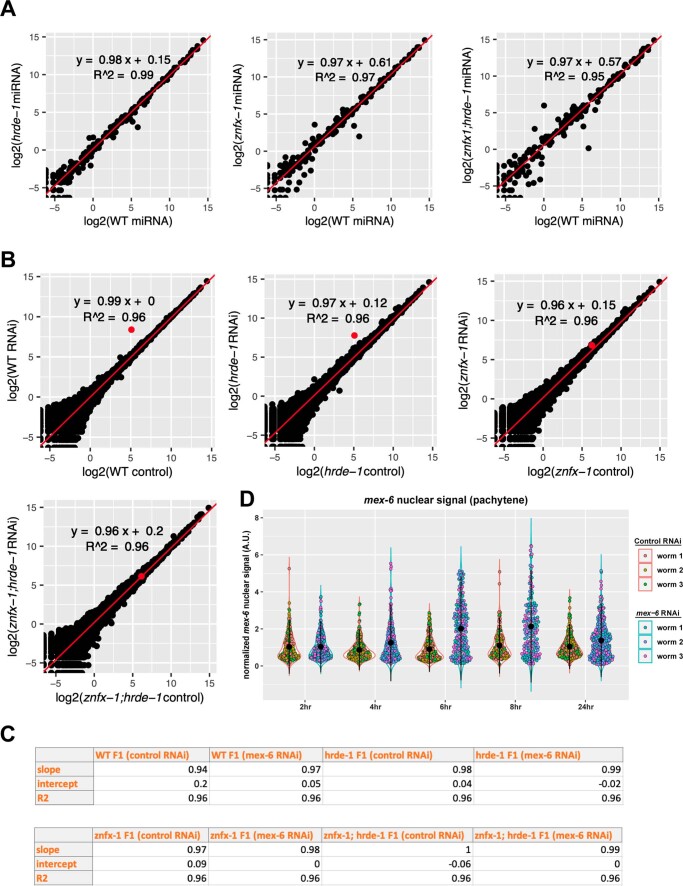
sRNAseq quality control analysis. **a**) Scatter plots comparing miRNA RPMs in wild-type (X-axis) and mutants (Y-axis) as indicated under control RNAi conditions. Linear regression is used to fit the data. miRNA counts in each condition are averaged across two replicates. **b**) Scatter plots comparing sRNA RPMs in the F1 progeny of control and *mex-6* RNAi fed P0 worms for the indicated genotypes. Each dot corresponds to a locus in the C. elegans genome. The red dot corresponds to the *mex-6* locus. Linear regression is calculated without *mex-6* sRNA counts. sRNA counts in each condition were averaged across two replicates. **c**) Linear regression statistics modelling the relationship between the two sRNAseq replicates for each genotype in both control and *mex-6* RNAi conditions. **d**) Super plot showing data from Fig. 2d with nuclei colour-coded to indicate worm origin. Central black dot and error bars represent the mean and standard deviation respectively. The distribution of values from each worm overlap. See legend for Fig. 2d for further description.

The number of miRNA reads were comparable between the wild-type and mutant libraries (Extended Data Fig. 9), suggesting that that there are no global changes in sRNA levels that could skew comparisons between genotypes. Comparisons of replicates confirmed the quality of each library (Extended Data Fig. 9).

For sRNA read-coverage analysis, mapped sRNA reads across the *mex-6* gene were placed into 5-bp bins. The number of nucleotides per bin were normalized to library size and averaged across two technical replicates. sRNAs present in the control RNAi condition (L4440 RNAi vector) were then subtracted from the RNAi condition. Scripts are available on request.

For the mRNAseq libraries, reads were aligned to the UCSC ce10 reference genome using HISAT2 (ref. ^68^). To assess the number of reads mapping to the *mex-5* and *mex-6* genes, the total number of reads mapping to these loci were counted and normalized to the number of singly aligned reads mapping to the entire genome (that is, library size) per million reads (RPM).

All high-throughput sequencing data were analysed on an Ubuntu 16.04.6 LTS (GNU/Linux 4.15.0-142-generic x86_64) computer.

### pUGylation assays

We synthesized pUG complementary DNA using the SuperScript III first-strand synthesis system (Thermo Fisher, cat. no. 18080051) according to the manufacturer’s instructions and stored it at −20 °C.

The pUG cDNA (1 µl) was inputted into a 20 µl GoTaq PCR reaction (Promega, cat no. M7123) with the first adaptor-specific primer (Shukla et al.^9^; OJPO398 in Supplementary Table 1) and the first gene-specific primer (‘f1’ primers in Supplementary Table 1). The samples were diluted 1:100 and 1 µl was added to a second 20 µl GoTaq PCR reaction with the second adaptor-specific primer (Shukla et al.^9^; OJPO399 in Supplementary Table 1) and the second gene-specific primer (‘f2’ primers in Supplementary Table 1). The reactions were run on a 1% agarose gel and imaged using a Typhoon imager for analysis of transcripts 500–1,000 bp in length.

For pUGylation assays in immunoprecipitated samples, 8 µl of 20 µl RNA eluted from the immunoprecipitation RNA extraction was used for the pUG cDNA synthesis (representative of approximately 40% of the immunoprecipitated RNA). RNA (5 µg) was used for the immunoprecipitation input pUG cDNA synthesis (representative of approximately 0.25% of the input RNA). Reverse transcription reactions were subjected to two rounds of PCR as described earlier.

### Immunoprecipitation

Filtered adult worms were washed in sonication buffer (20 mM Tris–HCl pH 7.5, 200 mM NaCl, 2.5 mM MgCl_2_, 10% glycerol, 0.5% NP-40 and 1 mM dithiothreitol) with cOmplete, Mini, EDTA-free protease inhibitor cocktail (Millipore Sigma, cat no. 11836170001; one tablet per 10 ml) and stored at −80 °C. The samples were thawed on ice with SUPERase•In RNase inhibitor (Thermo Fisher, cat no. AM2694; final concentration of 80 U ml^−1^), sonicated using a Branson Digital Sonifier SFX 250 with a microtip (15 s on, 45 s off, 20% power, 3 min in total), cleared through centrifugation at 18,400*g* and 4 °C for 15 min and the protein concentrations were determined using a Pierce BCA assay (Thermo Fisher, cat no. 23225). For the immunoprecipitation, 400 µl of 500 µg µl^−1^ lysate was added to 20 µl anti-FLAG M2 magnetic beads slurry (Millipore Sigma, cat no. M8823-1ML) that had been washed three times in 200 µl of sonication buffer + 80 U ml^−1^ SUPERase•In RNase inhibitor. An equivalent of 1% input lysate was used for analysis of the immunoprecipitation by western blotting (see the ‘Western blotting’ section). An additional equivalent of 50% of input lysate was saved for RNA extraction (see ‘RNA extraction and purification’). The samples were rotated at 4 °C for 2 h, cleared with a magnetic stand and 4.2 µl of the supernatant (approximately 1%) was saved for western analysis (see ‘Western blotting’). The beads were washed 5× with 500 µl of sonication buffer + 80 U ml^−1^ SUPERase•In RNase inhibitor and resuspended in 100 µl of sonication buffer + 80 U ml^−1^ SUPERase•In RNase inhibitor. A 1-µl volume of bead slurry (1%) was removed for western analysis of the immunoprecipitates (elution occurred through the addition of sample buffer and boiling; see ‘Western blotting’). TRIzol was added to the remaining elution/bead solution for RNA extraction (see ‘RNA extraction and purification’).

### Western blotting

Samples were resuspended in 200 mM dithiothreitol and 1×Tris-Glyc SDS sample buffer (Thermo Fisher, cat no. LC2676), flash-frozen and stored at −80 °C. The samples were heated to 95 °C for 10 min and run in Novex Tris–glycine SDS running buffer (Thermo Fisher, cat no. LC2675) on a Novex WedgeWell 6%, Tris–glycine, 1.0 mm, mini protein 12-well gel (Thermo Fisher, cat no. XP00062BOX) with a Spectra multicolor high range protein ladder (Thermo Fisher, cat no. 26625). The samples were transferred to an Immobilon-P PVDF membrane (Sigma-Aldrich, cat no. IPVH) and blocked in PBS containing 0.1% Tween 20 and 5% Blotting-grade blocker (BioRad, cat no. 1706404) for 30 min. The membranes were incubated overnight with primary antibody to FLAG M2 (1:500 dilution; Millipore Sigma, cat no. MF1804) in PBS containing 0.1% Tween 20 and 5% Blotting-Grade Blocker, washed three times (5–10 min) in PBS containing 0.1% Tween 20, incubated for 30 min with goat anti–mouse IgG1 horseradish peroxidase-conjugated secondary antibody (1:2,500 dilution; JacksonImmuno, cat no. 115-035-205) in PBS containing 0.1% Tween 20 and 5% Blotting-grade blocker at room temperature, washed another three times in PBS containing 0.1% Tween 20 and visualized with HyGLO quick spray chemiluminescent HRP antibody detection reagent (Denville Scientific Inc, cat no. E2400) and a KwikQuant imager (Kindle Biosciences, LLC, cat no. D1001).

### Quantitative PCR with reverse transcription analysis

Total RNA (500 ng) was used as input into a 10-µl reaction of a SuperScript VILO cDNA synthesis kit (Thermo Fisher, cat no. 11754050) according to the manufacturer’s instructions. A 3-µl volume of cDNA (1:20 dilution) was used in a 10 µl quantitative-PCR reaction using SsoAdvanced Universal SYBR Green Supermix (BioRad, cat no. 1725271) and 250 nM primers (Supplementary Table 1). Parallel *tbb-2* quantitative PCR reactions were run for each sample for normalization. The reactions were run on a QuantStudio 6 Flex real-time PCR system (Thermo Fisher, cat no. 4485691). Fold-change calculations were performed using the ΔΔ*C*_t_ method. Mean *tbb-2* *C*_t_ values were subtracted from the respective *mex-6*, *puf-5* and *oma-1* *C*_t_ values (Δ*C*_t_). Average Δ*C*_t_ values from the control condition of each genotype were then subtracted from the control and gene-specific RNAi condition of the same genotype (ΔΔ*C*_t_). Fold change with respect to the control condition was calculated using $2^{\Delta\Delta{C}_{\rm{t}}}$. Three technical replicates were run per sample.

### Microscopy

Fluorescence confocal microscopy was performed using a ×63, 1.4 numerical aperture objective on an inverted ZEISS LSM 880-AiryScan (Fig. 2e,f and Extended Data Figs. 2f, 5a,b) or inverted Zeiss Axio Observer with CSU-W1 Sora spinning disk scan head (Yokogawa), 1X/×2.8 relay lens (Yokogawa), fast piezo z-drive (Applied Scientific Instrumentation), a iXon Life 888 EMCCD camera (Andor) and a 405/488/561/637 nm solid-state laser (Coherent) with a 405/488/561/640 nm transmitting dichroic (Semrock) and 624–40/692–40/525–30/445–45 nm bandpass filter (Semrock; all other figures). The ZEISS ZEN 3.4 (blue edition) imaging software and Airyscan Processing were used for images captured with the AiryScan. The Slidebook v.6.0 software from Intelligent Imaging Innovations was used for images captured with the Zeiss Axio.

### Image analysis and quantification

All FISH experimental values were normalized across experiments using control RNA (typically *puf-5*) visualized by FISH in a second colour. Images (Zeiss Axio Observer) were processed in Fiji (https://imagej.net/software/fiji/downloads). All quantification was processed using R (version 4.1.0) and RStudio version 1.4.1717.

For quantification of the cytoplasmic signals, five worms were used for each condition. Regions of interest (ROIs) were drawn in single *z* planes, mean intensity values were calculated for each channel (*mex-6* (experimental) and *puf-5* (control)) and background mean intensity values measured in adjacent soma tissues were subtracted. The background-subtracted mean *mex-6* germline measurements were normalized to the background-subtracted mean *puf-5* germline measurement.

For quantification of the pachytene nuclear signals, maximum projections were taken from half of the *C. elegans* germline (in the *z*-direction) and individual ROIs were drawn around ten rows of pachytene nuclei, starting in the centre of the *mex-6-*expression region. The maximum, mean and median value for each ROI was measured in each channel. The median *mex-6* value for each nucleus was subtracted from its respective *mex-6* maximum value. The *mex-6* maximum value was then normalized by dividing it by the mean *puf-5* value measured for the respective ROI. The final equation was as follows:

$$y = \frac{{{\mathrm{maximum}}\,mex{\mbox{-}{\textit{6}}}\,{\mathrm{value}}\,{\mathrm{of}}\,{\mathrm{ROI}} - {\mathrm{median}}\,mex{\mbox{-}}{\textit{6}}\,{\mathrm{signal}}\,{\mathrm{of}}\,{\mathrm{ROI}}}}{{{\mathrm{mean}}\,puf{\mbox{-}{\textit{5}}}\,{\mathrm{signal}}\,{\mathrm{ROI}}}}$$

Values were plotted for three individual worms for each condition. Values across nuclei from different worms overlap (Extended Data Fig. 9d).

For quantification of the granule signals, ROIs for individual granules were drawn by masking in FIJI, and the mean *mex-6* and *puf-5* values were measured for each granule. Background mean intensity values were measured in adjacent soma tissues for both channels and subtracted from the measured values in the germline. The background-subtracted mean *mex-6* germline measurement was then normalized to the background-subtracted mean *puf-5* germline measurement and the values were plotted. Five individual worms were used for each condition.

To normalize the image display shown in Fig. 7b, the pixel-intensity distribution mean of the *tbb-2* RNA channel (used as a control for normalization) of the *znfx-1* mutant was adjusted to match that of the wild-type using ImageJ. The pUG RNA pixel-intensity distribution was then proportionally adjusted. Equally adjusted channels are also shown in Extended Data Fig. 8c.

### Statistics and reproducibility

For information regarding the statistical analysis used, see the figure legends for each graph. *P* values are indicated in each figure. Sample sizes were chosen based on an estimated number of samples that seemed to reflect the variability of the population. No statistical methods were used to pre-determine sample size. Data were only excluded from the analysis if the control feature (that is, *puf-5* RNA) used for normalizing between samples and conditions seemed to be aberrant (such instances were infrequent). The investigators were not blinded to allocation during experiments and outcome assessment. All in situ experiments were performed three or more times, with the exception of Extended Data Figs. 1c,d, 2b,e,h,i, 3b, 5c, which were all performed twice. The in situ experiments in Extended Data Figs. 3c and 4b were performed once. The mRNAseq from Extended Data Fig. 2c and quantitative-PCR data from Extended Data Fig. 6a–c were prepared from single biological replicates for each condition. The sRNAseq experiments were conducted in duplicate from a single biological source of RNA (Fig. 6a,b and Extended Data Fig. 6d–g). The pUGylation immunoprecipitation experiments were performed three independent times.

### Reporting summary

Further information on research design is available in the Nature Research Reporting Summary linked to this article.

## Online content

Any methods, additional references, Nature Research reporting summaries, source data, extended data, supplementary information, acknowledgements, peer review information; details of author contributions and competing interests; and statements of data and code availability are available at 10.1038/s41556-022-00940-w.

## Supplementary information

Reporting Summary

Supplementary Table Supplementary Table 1. List of oligonucleotides used in this study. Supplementary Table 2. FISH probe sequences used in this study.

## Data Availability

RNAseq datasets have been deposited onto the NCBI Sequence Read Archive (SRA) under the BioProject accession number PRJNA819556. Source data are provided with this paper. All other data supporting the findings of this study are available from the corresponding author on reasonable request.

## Source data

Source Data Fig. 2 Statistical source data.
Source Data Fig. 3 Statistical source data.
Source Data Fig. 4 Statistical source data.
Source Data Fig. 5 Statistical source data.
Source Data Fig. 6 Statistical source data.
Source Data Fig. 6 Unprocessed western blots and/or gels.
Source Data Fig. 7 Unprocessed western blots and/or gels.
Source Data Extended Data Fig. 2 Statistical source data.
Source Data Extended Data Fig. 3 Statistical source data.
Source Data Extended Data Fig. 4 Statistical source data.
Source Data Extended Data Fig. 5 Statistical source data.
Source Data Extended Data Fig. 6 Statistical source data.
Source Data Extended Data Fig. 7 Unprocessed western blots and/or gels.
Source Data Extended Data Fig. 8 Unprocessed western blots and/or gels.
