# Supplementary material for: The conserved helicase ZNFX-1 memorializes silenced RNAs in perinuclear condensates
Source: Nat Cell Biol. 2022 Jun 23;24(7):1129–40. doi: 10.1038/s41556-022-00940-w (PMC9276528; doi:10.1038/s41556-022-00940-w)
Supplement: Supplementary file 1 — Reporting Summary [file 41556_2022_940_MOESM1_ESM.pdf]

nature portfolio

Corresponding author(s): Geraldine Seydoux

Last updated by author(s): Mar 2, 2022

Reporting Summary

Nature Portfolio wishes to improve the reproducibility of the work that we publish. This form provides structure for consistency and transparency in reporting. For further information on Nature Portfolio policies, see our Editorial Policies and the Editorial Policy Checklist.

Statistics

For all statistical analyses, confirm that the following items are present in the figure legend, table legend, main text, or Methods section.

n/a	Confirmed	
☐	☒	The exact sample size (*n*) for each experimental group/condition, given as a discrete number and unit of measurement
☐	☒	A statement on whether measurements were taken from distinct samples or whether the same sample was measured repeatedly
☐	☒	The statistical test(s) used AND whether they are one- or two-sided<br>*Only common tests should be described solely by name; describe more complex techniques in the Methods section.*
☐	☒	A description of all covariates tested
☐	☒	A description of any assumptions or corrections, such as tests of normality and adjustment for multiple comparisons
☐	☒	A full description of the statistical parameters including central tendency (e.g. means) or other basic estimates (e.g. regression coefficient) AND variation (e.g. standard deviation) or associated estimates of uncertainty (e.g. confidence intervals)
☐	☒	For null hypothesis testing, the test statistic (e.g. *F*, *t*, *r*) with confidence intervals, effect sizes, degrees of freedom and *P* value noted<br>*Give P values as exact values whenever suitable.*
☒	☐	For Bayesian analysis, information on the choice of priors and Markov chain Monte Carlo settings
☒	☐	For hierarchical and complex designs, identification of the appropriate level for tests and full reporting of outcomes
☐	☒	Estimates of effect sizes (e.g. Cohen's *d*, Pearson's *r*), indicating how they were calculated

Our web collection on statistics for biologists contains articles on many of the points above.

Software and code

Policy information about availability of computer code

Data collection	Microscopy data were collected using 3i SlideBook6 and ZEISS ZEN 3.4 (blue edition).
Data analysis	Microscopy data were analyzed using FIJI software (v2.1.0/1.53f; Build 5f23140693), R (version 4.1.0), RStudio (version 1.4.1717) using custom scripts. Statistics and graphs were calculated/constructed in R.<br>High-throughput sequencing data were analyzed on an Ubuntu 16.04.6 LTS (GNU/Linux 4.15.0-142-generic x86_64) computer using Cutadapt (version 2.10) and HISAT2 (version 2.2.1).

For manuscripts utilizing custom algorithms or software that are central to the research but not yet described in published literature, software must be made available to editors and reviewers. We strongly encourage code deposition in a community repository (e.g. GitHub). See the Nature Portfolio guidelines for submitting code & software for further information.

Data

Policy information about availability of data

All manuscripts must include a data availability statement. This statement should provide the following information, where applicable:

- Accession codes, unique identifiers, or web links for publicly available datasets
- A description of any restrictions on data availability
- For clinical datasets or third party data, please ensure that the statement adheres to our policy

All data will be made available on request. RNA sequencing datasets have been deposited onto the NCBI Sequence Read Archive (SRA) under the BioProject accession number of PRJNA819556.

Field-specific reporting

Please select the one below that is the best fit for your research. If you are not sure, read the appropriate sections before making your selection.

☒ Life sciences ☐ Behavioural & social sciences ☐ Ecological, evolutionary & environmental sciences

For a reference copy of the document with all sections, see nature.com/documents/nr-reporting-summary-flat.pdf

Life sciences study design

All studies must disclose on these points even when the disclosure is negative.

Sample size	Sample sizes were chosen based off of an estimated number of samples that appeared to reflect the variability of the population. No statistical method was used to predetermine sample size.
Data exclusions	Data were only excluded from the analysis if the control feature used for normalizing between samples and conditions appeared aberrant. Such instances were infrequent.
Replication	Experiments were conducted multiple times using RNAi triggers against three different genes. Similar observations were made for these three triggers.
Randomization	Samples were allocated into experimental groups by genotype and RNAi condition. Covariates were controlled for by maintaining all samples in the same growth and media conditions.
Blinding	Investigators were not blinded for these experiments, as the identity of each condition could always be identified through examination of the RNA level alone.

Reporting for specific materials, systems and methods

We require information from authors about some types of materials, experimental systems and methods used in many studies. Here, indicate whether each material, system or method listed is relevant to your study. If you are not sure if a list item applies to your research, read the appropriate section before selecting a response.

Materials & experimental systems

n/a	Involved in the study	
☐	☒	Antibodies
☒	☐	Eukaryotic cell lines
☒	☐	Palaeontology and archaeology
☐	☒	Animals and other organisms
☒	☐	Human research participants
☒	☐	Clinical data
☒	☐	Dual use research of concern

Methods

n/a	Involved in the study	
☒	☐	ChIP-seq
☒	☐	Flow cytometry
☒	☐	MRI-based neuroimaging

Antibodies

Antibodies used	anti-FLAG M2 primary antibody (Millipore Sigma; Cat #: MF1804)<br>Goat Anti-Mouse IgG1 HRP-conjugated secondary antibody (JacksonImmuno; Cat #: 115-035-205)
Validation	Antibodies were self validated via western blot on a strain lacking the epitope, and no bands were detected (see Fig. S8A).

Animals and other organisms

Policy information about studies involving animals; ARRIVE guidelines recommended for reporting animal research

Laboratory animals	Experiments were conducted on adult C. elegans hermaphrodites, embryos, and L1s. All strains were of the N2 background.
Wild animals	No wild animals were used in this study.
Field-collected samples	No field-collected samples were used in this study.
Ethics oversight	No ethical approval or guidance is needed for C. elegans work.

Note that full information on the approval of the study protocol must also be provided in the manuscript.
