# Supplementary figures and images for: The conserved helicase ZNFX-1 memorializes silenced RNAs in perinuclear condensates
Source: Nat Cell Biol. 2022 Jun 23;24(7):1129–40. doi: 10.1038/s41556-022-00940-w (PMC9276528; doi:10.1038/s41556-022-00940-w)

6C

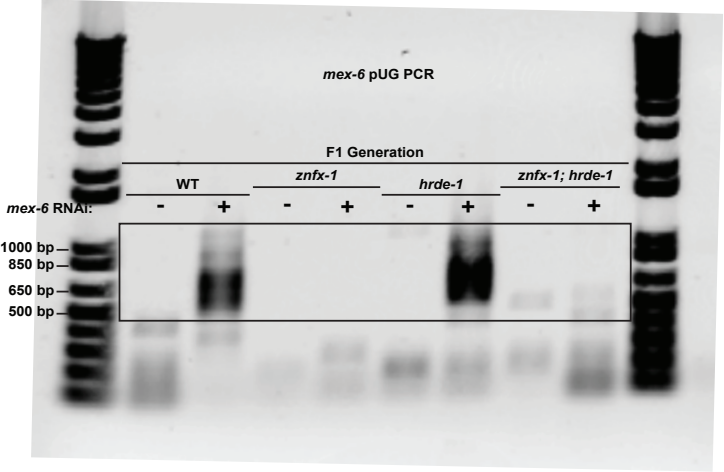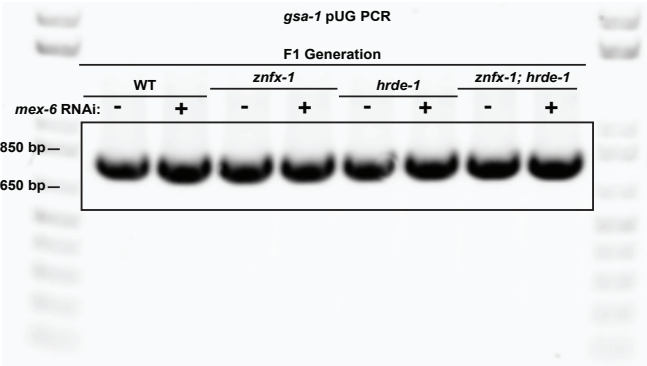

6D

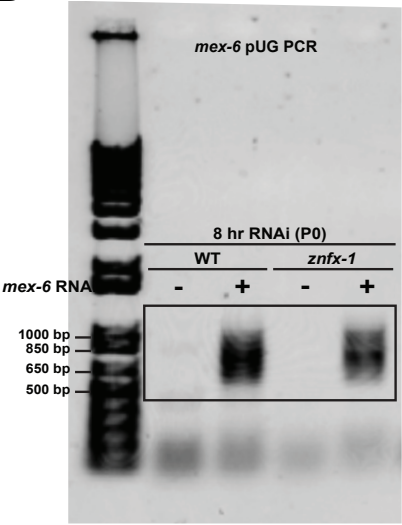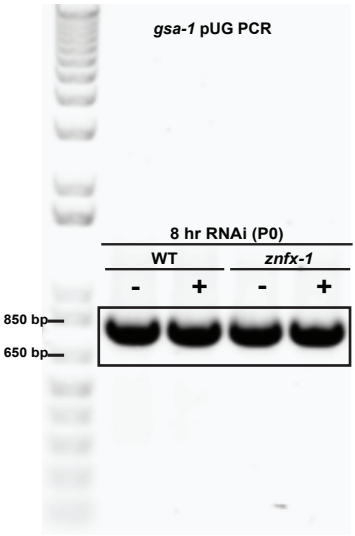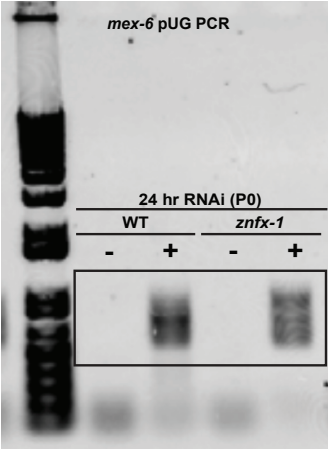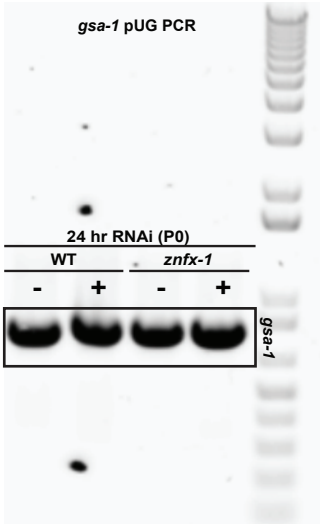

6E

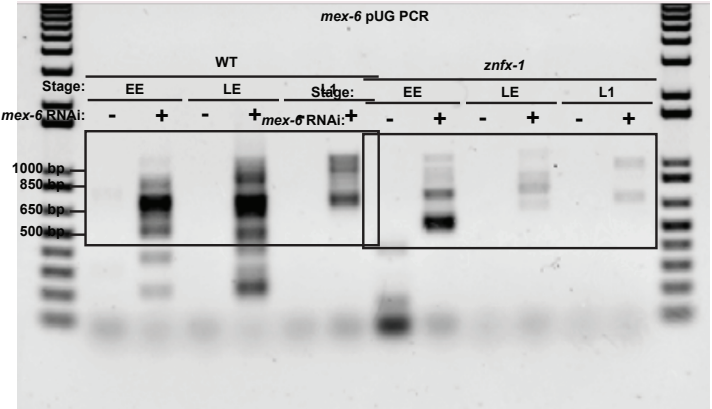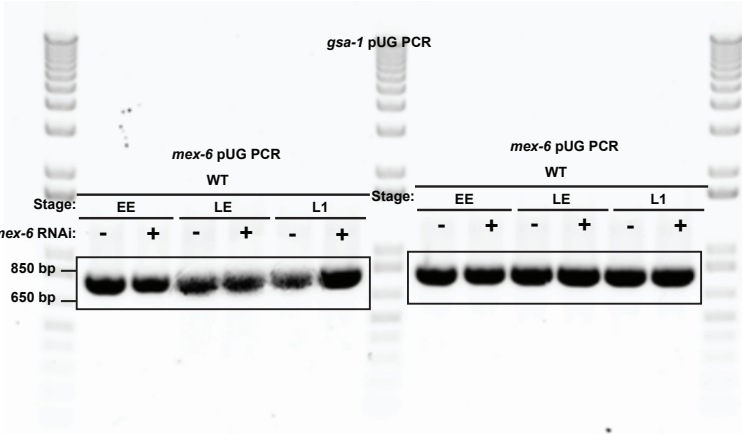

Supplement: Source Data Fig. 6 — Unprocessed western blots and/or gels. [file 41556_2022_940_MOESM8_ESM.pdf]

7C

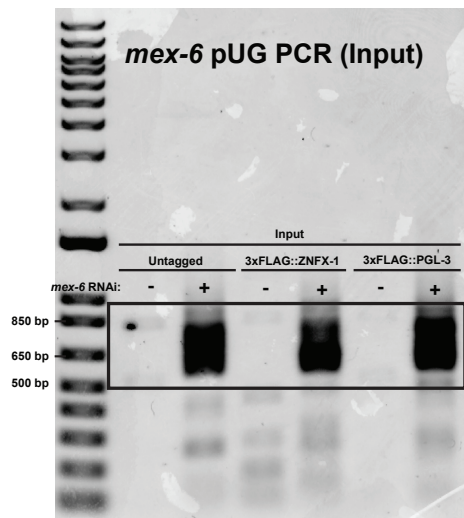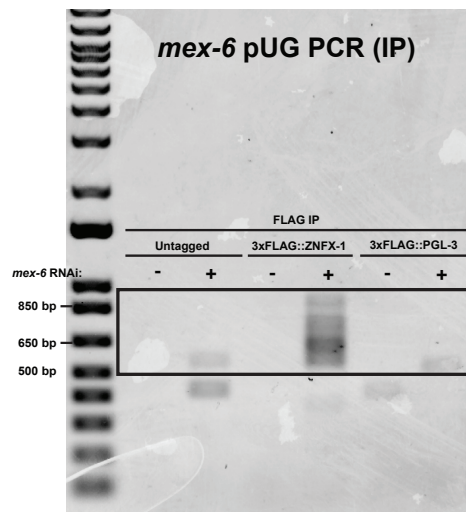

Supplement: Source Data Fig. 7 — Unprocessed western blots and/or gels. [file 41556_2022_940_MOESM9_ESM.pdf]

**S7A**

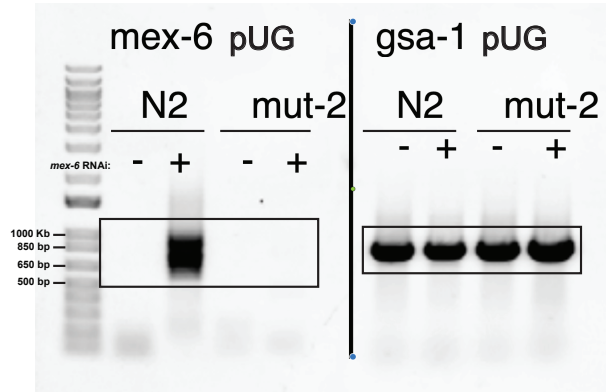

**S7B,C**

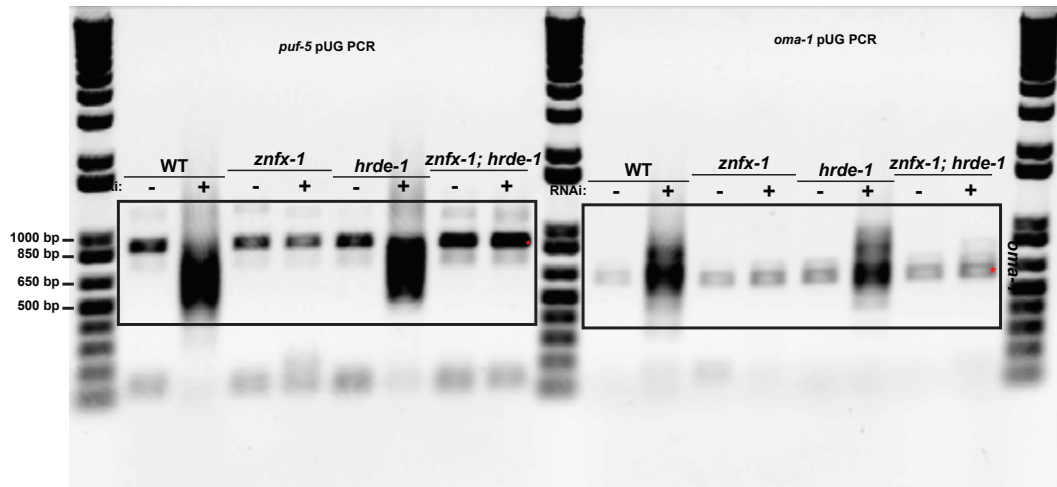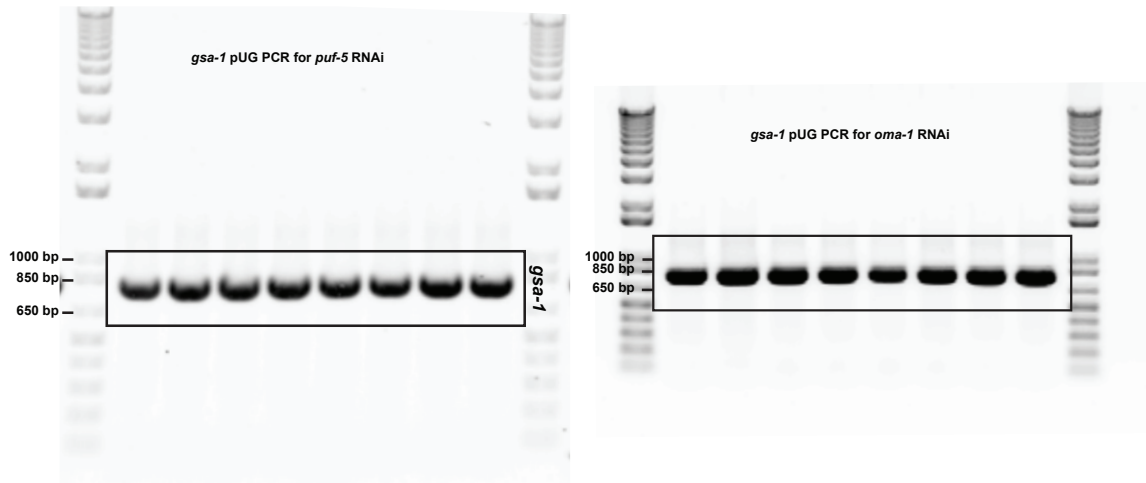

Supplement: Source Data Extended Data Fig. 7 — Unprocessed western blots and/or gels. [file 41556_2022_940_MOESM15_ESM.pdf]

S8A

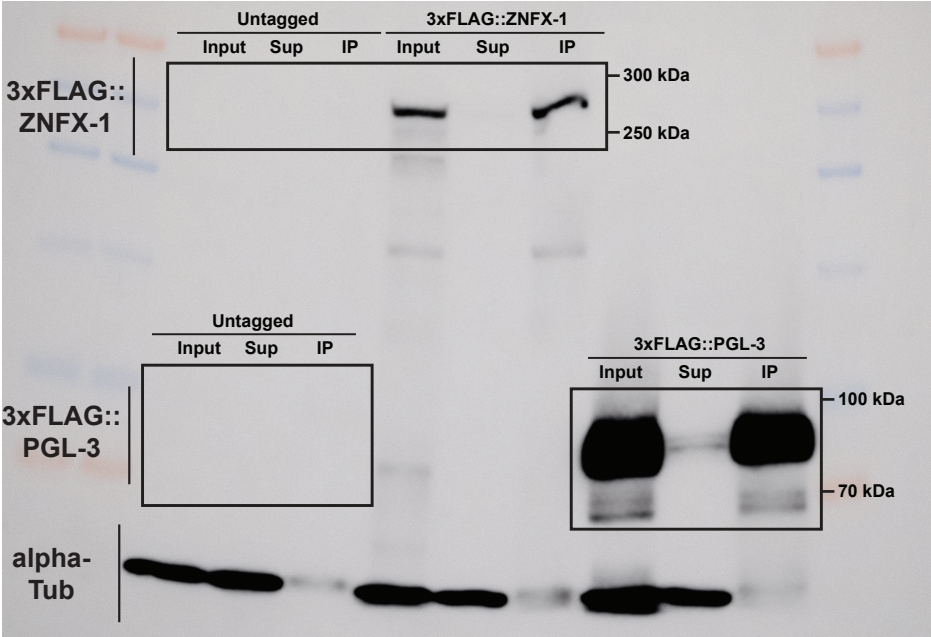

S8B

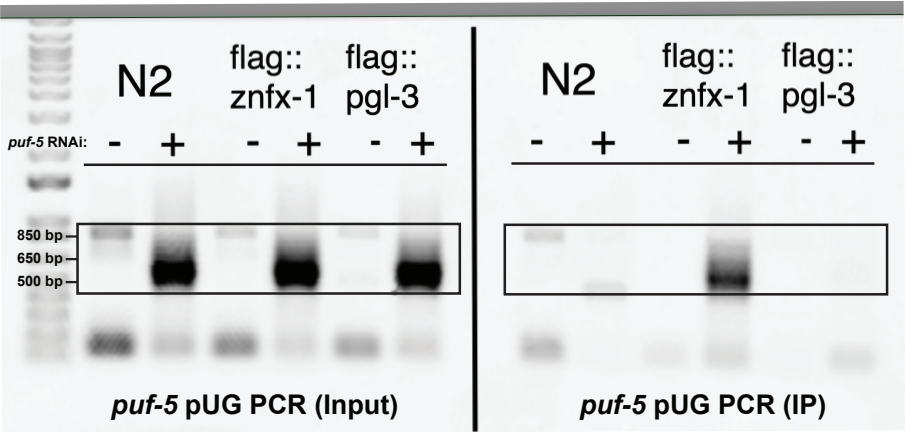

Supplement: Source Data Extended Data Fig. 8 — Unprocessed western blots and/or gels. [file 41556_2022_940_MOESM16_ESM.pdf]
